# Structure-Based Design, Synthesis, Biological Evaluation, and Molecular Docking of Novel PDE10 Inhibitors With Antioxidant Activities

**DOI:** 10.3389/fchem.2018.00167

**Published:** 2018-05-15

**Authors:** Jinxuan Li, Jing-Yi Chen, Ya-Lin Deng, Qian Zhou, Yinuo Wu, Deyan Wu, Hai-Bin Luo

**Affiliations:** School of Pharmaceutical Sciences, Sun Yat-sen University, Guangzhou, China

**Keywords:** Phosphodiesterase-10A, papaverine, antioxidant activity, Alzheimer's disease, molecular docking

## Abstract

Phosphodiesterase 10 is a promising target for the treatment of a series of central nervous system (CNS) diseases. Imbalance between oxidative stress and antioxidant defense systems as a universal condition in neurodegenerative disorders is widely studied as a potential therapy for CNS diseases, such as Alzheimer's disease (AD), Parkinson's disease (PD) and amyotrophic lateral sclerosis (ALS). To discover multifunctional pharmaceuticals as a treatment for neurodegenerative diseases, a series of quinazoline-based derivatives with PDE10 inhibitory activities and antioxidant activities were designed and synthesized. Nine out of 13 designed compounds showed good PDE10 inhibition at the concentration of 1.0 μM. Among these compounds, eight exhibited moderate to excellent antioxidant activity with ORAC (oxygen radical absorbance capacity) value above 1.0. Molecular docking was performed for better understanding of the binding patterns of these compounds with PDE10. Compound **11e**, which showed remarkable inhibitory activity against PDE10 and antioxidant activity may serve as a lead for the further modification.

## Introduction

Phosphodiesterases (PDEs) are a super enzyme family in charge of hydrolyzing the intracellular second messenger molecules 3′,5′-cyclic adenosine monophosphate (cAMP) and 3′,5′-cyclic guanosine monophosphate (cGMP) by degrading their phosphodiester bonds (Liu et al., 2001; Mehats et al., 2002; Castro et al., 2005; Bender and Beavo, 2006; Conti and Beavo, 2007; Houslay, 2010). As both cAMP and cGMP are involved in various extracellular signals and biological processes, the inhibition of PDEs can improve abnormal physiological processes caused by the low concentration of cAMP and/or cGMP by inhibiting their degradation (Lugnier, 2006; Francis et al., 2011). Thus, PDEs have been considered as promising targets for various diseases. Currently, up to 12 PDEs inhibitors have been approved, including PDE5 inhibitor sildenafil for erectile dysfunction and pulmonary arterial hypertension, PDE4 inhibitor roflumilast for chronic obstructive pulmonary disease (Sung et al., 2003; Christie, 2005). PDEs are classified into 11 distinct families (PDE1-11) based on the amino acid sequences, substrate specificities, and pharmacological properties (Bender and Beavo, 2006). The different expression of each subfamily on the organs and tissues makes specific PDE inhibitors have different therapeutic effects.

Phosphodiesterase 10 (PDE10) is a dual-specificity superfamily responsible for hydrolyzing both cAMP (*K*_*m*_ = 0.05 μM) and cGMP (*K*_*m*_ = 3 μM) (Soderling et al., 1999), which is highly expressed in the brain and has been considered as a potential target for the treatment of several central nervous system (CNS) disorders such as Schizophrenia and Huntington's disease (Hebb et al., 2004). Recent work has shown that blockade of PDE10A with selective inhibitors increases striatal cGMP and phosphorylated cAMP-response element binding protein (CREB), a downstream marker of cAMP production (Siuciak et al., 2006b). PDE10 inhibitors regulate the levels of cAMP and cGMP and activate the downstream dopaminergic pathways and glutamatergic pathways, which may avoid side effects of extrapyramidal system (EPS) caused by current anti-Schizophrenia drugs. In conditioned avoidance responding (CAR), an animal model predictive of drug antipsychotic activity, PDE10A inhibitors exhibited a dose-dependent inhibition. (Jones et al., 2015; Suzuki et al., 2015). Great efforts have been devoted in the development of PDE10 inhibitors in the last decade. Up to 7 candidates such as **MP-10** and **TAK-063** have entered the preclinical or clinical trials (Kehler, 2013; Gentzel et al., 2015; Wilson et al., 2015). However, there is still no PDE10 inhibitor approved on the market as a drug.

Oxidative stress (OS) has been suggested as a possible element in the pathogenesis of neurodegenerative disorders (Ceballos et al., 1990; Islam, 2017). Researches showed that neurodegenerative disorders are qualified by different levels of oxidative stress biomarkers and antioxidant defense biomarkers in the brain and peripheral tissues. Recently, some pharmaceuticals on the market with anti-oxidant activities have been demonstrated to decelerate neurodegenerative processes and enhance comprehension ability of the Oxidative stress (OS) characteristics in the pathobiology of these stubborn conditions (Mecocci and Polidori, 2012; Danta and Piplani, 2014). Moreover, experimental studies have proved the presence of elevated levels of Oxidative stress (OS) biomarkers accompanied with the impairments to antioxidant defenses in central and peripheral tissues in pathological process of Parkinson's disease (PD), Alzheimer's disease (AD), and amyotrophic lateral sclerosis (ALS). Pharmaceuticals with antioxidant activity enable biomarkers of the oxidant/antioxidant to rebalance in animal models, thus are widely studied as possible anti-neurodegenerative agents (Zhang et al., 2006; Niedzielska et al., 2016). Vinpocetine, a moderate PDE1 inhibitor with antioxidant activity, can significantly improve the learning and memory in the streptozotocin infused AD rat models. Vinpocetine acts as a neuroprotective agent, which is widely applied to the treatment of CNS disorders with good antioxidant activity and the observed cognitive effects and memory improvement of vinpocetine is believed to be bound up with the antioxidant mechanism and elevations of cGMP levels (Hindmarch et al., 1991; Bönöczk et al., 2000). As noted above, PDE inhibitors with antioxidant activities have potential possibility to apply in the treatment of several CNS disorders.

Till now, compounds with both PDE10A inhibitory activities and antioxidant activities have seldom been reported. Taking all these into consideration, a strategy to design lead compounds combining the pharmacophore of PDE10A inhibitors and antioxidants seems to be attractive and challenging. In this study, a series of compounds expected to exhibit both PDE10A inhibition and antioxidant activity were designed and synthesized based on the chemical structure of a natural derivative papaverine. Five compounds showed moderate to good PDE10A inhibitory activities. Compound **11e** showed good antioxidant activity as well as PDE10A inhibitory activity.

## Materials and methods

All starting materials and reagents were purchased from commercial suppliers (Adamas, Energy, Bide, Sigma-Aldrich, ShuYa, J&K, and Meryer) and used directly without further purification. Chemical HG/T2354-92 silica gel (200–300 mesh, Haiyang®) was used for chromatography, and silica gel plates with fluorescence F254 (0.25 mm, Huanghai®) were used for thin-layer chromatography (TLC) analysis. Reactions requiring anhydrous conditions were performed under argon or a calcium chloride tube. ^1^H NMR and ^13^C NMR spectra were recorded at room temperature on a Bruker AVANCE III 400 instrument with tetramethylsilane (TMS) as an internal standard (Presentation 1). The following abbreviations are used: s (singlet), d (doublet), t (triplet), m (multiplet), dd (doublet of doublets), dt (doublet of triplets), td (triplet of doublets), and br (broad signal). Coupling constants were reported in Hz. Low- and high-resolution mass spectra (LRMS and HRMS) were recorded on a MAT-95 spectrometer. The purity of compounds was determined by reverse-phase high-performance liquid chromatography (HPLC) analysis confirming to be over 95%. HPLC instrument: SHIMADZU LC-20AT (column: Hypersil BDS C_18_, 5.0 μm, 4.6 × 150 mm (Elite); Detector: SPD-20A UV/VIS detector, UV detection at 254 nm; Elution, MeOH in water (60–80%, v/v); *T* = 25°C; and flow rate = 0.8–1.0 mL/min.

### 7-methoxy-4-oxo-3,4-dihydroquinazolin-6-yl acetate (4)

Pyridine (4 mL) was added dropwise to the solution of 6-hydroxy-7-methoxyquinazolin-4(3H)-one **3** (1.92 g, 10.0 mmol) in acetic anhydrate (20 mL). The reaction mixture was heated at 100°C for 2 h and then cooled to room temperature. After the mixture was poured into ice water, a white solid was precipitated. The precipitate was collected, washed with water and dried to give the compound **4** (2.32 g, 99%) as a white solid. ^1^H NMR (400 MHz, DMSO – *d*_6_) δ 8.09 (s, 1H), 7.76 (s, 1H), 7.28 (s, 1H), 3.92 (s, 3H), 2.30 (s, 3H).

### 4-chloro-7-methoxyquinazolin-6-yl acetate (5)

To a solution of **4** (2.34 g, 10.0 mmol) in SOCl_2_ (20 mL) was added DMF (0.1 mL) dropwise. The mixture was stirred at 80°C for 2.5 h and then concentrated under vacuum, providing compound **5** (2.22 g, 88%) which could be used in the next step without further purification. ^1^H NMR (400 MHz, DMSO – *d*_6_) δ 9.02 (s, 1H), 8.02 (s, 1H), 7.65 (s, 1H), 4.03 (s, 4H), 2.36 (s, 3H).

### 7-methoxy-4-morpholinoquinazolin-6-ol (7)

A solution of compound **5** (2.52 g, 10.0 mmol) and morpholine (1.04 g, 12.0 mmol) in DMF (20 mL) was stirred at 80°C for 6 h. The mixture was then poured into the ice water and a white solid was precipitated, which was collected and washed with ice water to afford compound **6**. The compound **6** was dissolved in methanol (20 mL). Ammonia (2.5 mL) was added to the mixture and the mixture was then stirred under reflux for 2 h. The solvents were evaporated under vacuum. The crude product was recrystallized using methanol to afford compound **7** (1.87 g, 72%). ^1^H NMR (400 MHz, DMSO – *d*_6_) δ 8.52 (s, 1H), 7.24 (s, 1H), 7.21 (s, 1H), 3.93 (s, 3H), 3.83 – 3.75 (m, 4H), 3.54 – 3.47 (m, 4H).

### 4-(6-(2-(1H-indol-3-yl)ethoxy)-7-methoxyquinazolin-4-yl) morpholine (8a)

To a solution of compound **7** (522 mg, 2.0 mmol) in DMF (20 mL) was added 3-(2-bromoethyl)-1H-indole (538 mg, 2.4 mmol) and potassium carbonate (690 mg, 5.0 mmol). The reaction mixture was refluxed for 3 h. After cooling to room temperature, adding water to quench the mixture, and then the residue was diluted with CH_2_Cl_2_ (30 mL) and washed with saturated aqueous sodium bicarbonate and water. The organic layer was dried over anhydrous sodium sulfate, and purified by silica gel column chromatography (petroleum ether/EtOAc, 3:1–1:1) to afford the title compound as a yellow solid. Purity: 97%; yield: 20%; ^1^H NMR (400 MHz, CDCl_3_) δ 8.67 (s, 1H), 8.29 (s, 1H), 7.69 (d, *J* = 7.8 Hz, 1H), 7.38 (d, *J* = 8.0 Hz, 1H), 7.22 (t, *J* = 7.5 Hz, 1H), 7.17 (s, 1H), 7.15 (t, *J* = 7.4 Hz, 1H), 7.08 (s, 1H), 4.37 (t, *J* = 7.0 Hz, 2H), 4.02 (s, 3H), 3.82 (s, 4H), 3.60 (s, 4H), 3.39 (t, *J* = 7.0 Hz, 2H); ^13^C NMR (101 MHz, CDCl_3_) δ 163.76, 155.11, 152.96, 149.17, 148.04, 136.26, 127.46, 122.49, 122.22, 119.51, 118.76, 111.85, 111.42, 111.28, 107.65, 104.44, 69.48, 66.63 × 2, 56.19, 50.21 × 2, 25.19; LRMS (ESI) m/z [M+H]^+^ 405.2; HRMS (ESI) m/z calcd C_23_H_24_N_4_O_3_ [M+H]^+^ 405.1927, found 405.1922.

### General procedure for synthesis of compounds 8b-8c

To a solution of compound **7** (522 mg, 2.0 mmol) in DMF (20 mL) was added the EDCI (575 mg, 3.0 mmol) and DMAP (12 mg, 0.1 mmol). The reaction mixture was stirred at room temperature for 0.5 h, and then the corresponding acid (2.4 mmol) was added, the reaction mixture was refluxed overnight. After cooling to room temperature, adding water to quench the mixture, and then the residue was diluted with CH_2_Cl_2_ (30 mL) and washed with saturated aqueous sodium bicarbonate and water. The organic layer was dried over anhydrous sodium sulfate, and purified by silica gel column chromatography (petroleum ether/EtOAc, 3:1 to 1:1) to afford the title compound as a white solid.

### 7-methoxy-4-morpholinoquinazolin-6-yl 3-(4-hydroxyphenyl)acrylate (8b)

White solid; purity: 97%; yield: 10%; ^1^H NMR (400 MHz, CDCl_3_) δ 8.70 (s,1H), 7.86 (d, *J* = 16.0 Hz, 1H), 7.61 (s, 1H), 7.52 (d, *J* = 7.5 Hz, 2H), 7.35 (s, 1H), 6.90 (d, *J* = 7.5 Hz, 2H), 6.54 (d, *J* = 15.7 Hz, 1H), 5.35 (br, 1H), 3.96 (s, 3H), 3.91–3.85 (m, 4H), 3.78–3.73 (m, 4H); ^13^C NMR (101 MHz, CDCl_3_) δ 177.80, 164.07, 155.98, 154.20, 151.68, 147.48, 139.24, 138.32, 130.45 × 2, 117.87, 116.23 × 2, 113.08, 110.55, 108.16, 94.86, 66.76 × 2, 56.28, 50.18 × 2; HRMS (ESI) m/z calcd C_22_H_21_N_3_O_5_ [M+H]^+^ 408.1559, found 408.1554.

### 7-methoxy-4-morpholinoquinazolin-6-yl 5-(1,2-dithiolan-3-yl)pentanoate (8c)

White solid; purity: 97%; yield: 16%; ^1^H NMR (400 MHz, CDCl_3_) δ 8.66 (s, 1H), 7.48 (s, 1H), 7.31 (s, 1H), 3.93 (s, 3H), 3.90–3.78 (m, 4H), 3.75–3.64 (m, 4H), 3.59 (dt, *J* = 12.9, 6.4 Hz, 1H), 3.14 (dtd, *J* = 17.9, 11.4, 6.8 Hz, 2H), 2.63 (t, *J* = 7.3 Hz, 2H), 2.46 (td, *J* = 12.4, 6.4 Hz, 1H), 1.91 (td, *J* = 13.7, 7.0 Hz, 1H), 1.76 (ddd, *J* = 29.4, 14.5, 8.2 Hz, 4H), 1.58 (ddd, *J* = 22.9, 14.4, 8.1 Hz, 2H); ^13^C NMR (101 MHz, CDCl_3_) δ 177.02, 155.73, 153.99, 151.77, 145.91, 139.05, 117.65, 111.54, 106.70, 66.68 × 2, 56.36, 50.08 × 2, 40.19, 38.45, 34.62, 34.29, 33.71, 28.81, 24.78; HRMS (ESI) m/z calcd C_21_H_27_N_3_O_4_S_2_ [M+H]^+^ 450.1521, found 450.1534.

### 4-chloro-6,7-dimethoxyquinazoline (10)

To a solution of **9** (10.0 mmol) in SOCl_2_ (20 mL) was added DMF (0.1 mL) dropwise. The mixture was stirred at 80°C for 2.5 h and then concentrated under vacuum, providing compound **10** (2.22 g, 90%) which could be used in the next step without further purification. ^1^H NMR (400 MHz, DMSO – *d*_6_) δ 8.88 (s, 1H), 7.46 (s, 1H), 7.40 (s, 1H), 4.01 (d, *J* = 6.0 Hz, 6H).

### General procedure for synthesis of compounds 11a-11h, 12 and 13

To a solution of **10** (2.0 mmol), the corresponding amine (3.0 mmol) in isopropanol (20 mL) was added triethylamine (6.0 mmol) dropwise. The reaction mixture was refluxed for 4 h and then concentrated under vacuum, providing crude product. The crude product was purified by silica gel column chromatography (CH_2_Cl_2_/MeOH, 100:1–40:1) to afford the title compound as a white solid.

### N-(2-(1H-indol-3-yl)ethyl)-6,7-dimethoxyquinazolin-4-amine (11a)

White solid; purity: 97%; yield: 60%; ^1^H NMR (400 MHz, DMSO–*d*_6_) δ 10.92 (s, 1H), 10.27 (br, 1H), 8.80 (s, 1H), 8.06 (s, 1H), 7.63 (d, *J* = 7.8 Hz, 1H), 7.35 (d, *J* = 8.1 Hz, 1H), 7.26 (d, *J* = 11.6 Hz, 2H), 7.07 (t, *J* = 7.5 Hz, 1H), 6.98 (t, *J* = 7.4 Hz, 1H), 3.97 (s, 1H), 3.95 (s, 7H), 3.93–3.91 (m, 1H), 3.13 (t, *J* = 7.4 Hz, 2H); ^13^C NMR (101 MHz, DMSO–*d*_6_) δ 159.44, 156.13, 150.20, 149.28, 136.72, 134.53, 127.62, 123.40, 121.49, 118.80, 114.08, 111.92, 111.57, 107.09, 104.42, 99.94, 57.29, 56.77, 42.80, 24.94; HRMS (ESI) m/z calcd C_20_H_20_N_4_O_2_ [M+H]^+^ 349.1665, found 349.1670.

### N-(1-(1H-indol-3-yl)propan-2-yl)-6,7-dimethoxyquinazolin-4-amine (11b)

White solid; purity: 96%; yield: 55%; ^1^H NMR (400 MHz, CDCl_3_) δ 8.59 (s, 1H), 8.37 (br, 1H), 7.70 (d, *J* = 7.8 Hz, 1H), 7.41 (d, *J* = 8.1 Hz, 1H), 7.28 (s, 1H), 7.21 (t, *J* = 7.5 Hz, 1H), 7.12 (t, *J* = 7.2 Hz, 2H), 6.56 (s, 1H), 5.74 (br, 1H), 4.96–4.85 (m, 1H), 3.97 (s, 3H), 3.75 (s, 3H), 3.24 (dd, *J* = 14.5, 5.9 Hz, 1H), 3.13 (dd, *J* = 14.4, 4.6 Hz, 1H), 1.40 (d, *J* = 6.4 Hz, 3H); ^13^C NMR (101 MHz, CDCl_3_) δ 157.73, 154.22, 154.00, 148.80, 146.09, 136.30, 128.25, 123.26, 122.12, 119.79, 118.90, 111.41, 111.28, 108.60, 107.39, 99.58, 56.13, 56.08, 46.72, 31.11, 20.06; HRMS (ESI) m/z calcd C_21_H_22_N_4_O_2_ [M+H]^+^ 363.1821, found 363.1806.

### 6,7-dimethoxy-N-(2-(5-methoxy-1H-indol-3-yl)ethyl)quinazolin-4-amine (11c)

White solid; purity: 97%; yield: 62%; ^1^H NMR (400 MHz, DMSO–*d*_6_) δ 10.67 (br, 1H), 8.41 (s, 1H), 8.10 (t, *J* = 5.5 Hz, 1H), 7.59 (s, 1H), 7.24 (d, *J* = 8.7 Hz, 1H), 7.17 (d, *J* = 2.3 Hz, 1H), 7.13 (d, *J* = 2.4 Hz, 1H), 7.11 (s, 1H), 6.72 (dd, *J* = 8.7, 2.4 Hz, 1H), 3.89 (d, *J* = 9.2 Hz, 6H), 3.88 (s, 3H), 3.81 (dd, *J* = 14.3, 6.3 Hz, 2H), 3.71 (s, 3H), 3.10–3.00 (m, 2H); ^13^C NMR (101 MHz, DMSO–*d*_6_) δ 158.27, 153.68, 153.60, 152.96, 148.25, 145.80, 131.37, 127.70, 123.27, 112.00, 111.86, 111.10, 108.56, 106.91, 101.99, 100.20, 55.97, 55.62, 55.17, 41.43, 24.85; HRMS (ESI) m/z calcd C_21_H_22_N_4_O_3_ [M+H]^+^ 379.1770, found 379.1766.

### 6,7-dimethoxy-N-(1-(5-methoxy-1H-indol-3-yl)propan-2-yl)quinazolin-4-amine (11d)

White solid; purity: 98%; yield: 51%; ^1^H NMR (400 MHz, CDCl_3_) δ 8.61 (s, 1H), 8.23 (br, 1H), 7.31 (d, *J* = 8.8 Hz, 1H), 7.20 (s, 1H), 7.14 (d, *J* = 1.9 Hz, 1H), 7.10 (s, 1H), 6.87 (dd, *J* = 8.8, 2.1 Hz, 1H), 6.47 (s, 1H), 5.41 (br, 1H), 4.91 (dt, *J* = 12.5, 6.4 Hz, 1H), 3.99 (s, 3H), 3.73 (s, 3H), 3.70 (s, 3H), 3.69–3.64 (m, 1H), 3.22 (dd, *J* = 14.5, 6.0 Hz, 1H), 3.08 (dd, *J* = 14.4, 4.1 Hz, 1H), 1.38 (d, *J* = 6.5 Hz, 3H); ^13^C NMR (101 MHz, CDCl_3_) δ 157.73, 154.25, 154.02, 148.91, 146.18, 131.36, 128.62, 124.05, 112.49, 112.18, 110.96, 108.61, 107.43, 100.45, 99.50, 56.14, 55.92, 55.52, 46.61, 31.10, 19.92; HRMS (ESI) m/z calcd C_22_H_24_N_4_O_3_ [M+H]^+^ 393.1927, found 393.1916.

### 3-(2-((6,7-dimethoxyquinazolin-4-yl)amino)ethyl)-1H-indol-5-ol (11e)

White solid; purity: 97%; yield: 62%; ^1^H NMR (400 MHz, DMSO–*d*_6_) δ 10.58 (s, 1H), 10.23 (t, *J* = 5.0 Hz, 1H), 8.79 (s, 1H), 8.71 (s, 1H), 8.09 (s, 1H), 7.30 (s, 1H), 7.14 (d, *J* = 8.5 Hz, 2H), 6.98 (d, *J* = 2.0 Hz, 1H), 6.62 (dd, *J* = 8.6, 2.2 Hz, 1H), 3.95 (d, *J* = 4.5 Hz, 6H), 3.90 (dd, *J* = 14.5, 6.7 Hz, 2H), 3.06–3.02 (m, 2H); ^13^C NMR (101 MHz, DMSO–*d*_6_) δ 159.03, 154.98, 152.15, 151.72, 150.69, 149.35, 131.28, 128.41, 123.65, 112.17, 111.80, 111.13, 108.23, 104.32, 103.27, 102.82, 56.79, 56.41, 42.20, 25.32; HRMS (ESI) m/z calcd C_20_H_20_N_4_O_3_ [M+H]^+^ 365.1614, found 365.1611.

### N-(2-(6-fluoro-1H-indol-3-yl)ethyl)-6,7-dimethoxyquinazolin-4-amine (11f)

White solid; purity: 97%; yield: 44%; ^1^H NMR (400 MHz, DMSO–*d*_6_) δ 10.90 (br, 1H), 8.39 (s, 1H), 8.09 (t, *J* = 5.5 Hz, 1H), 7.62 (dd, *J* = 8.6, 5.5 Hz, 1H), 7.59 (s, 1H), 7.21 (d, *J* = 2.1 Hz, 1H), 7.12 (dd, *J* = 11.1, 3.2 Hz, 2H), 6.85 (ddd, *J* = 9.8, 8.8, 2.3 Hz, 1H), 3.90 (s, 3H), 3.88 (s, 3H), 3.79 (dd, *J* = 14.3, 6.3 Hz, 2H), 3.08–3.02 (m, 2H); ^13^C NMR (101 MHz, DMSO–*d*_6_) δ 158.75, 154.27, 153.81, 148.81, 145.57, 136.57, 124.68, 123.73, 119.88, 112.71, 108.88, 107.32, 107.08, 102.48, 97.92, 97.67, 56.50, 56.17, 41.87, 25.22; HRMS (ESI) m/z calcd C_20_H_19_FN_4_O_2_ [M+H]^+^ 367.1570, found 367.1766.

### 6,7-dimethoxy-N-(2-(5-methyl-1H-indol-3-Yl)ethyl)quinazolin-4-amine (11g)

White solid; purity: 97%; yield: 68%; ^1^H NMR (400 MHz, DMSO–*d*_6_) δ 10.67 (br, 1H), 8.39 (s, 1H), 8.05 (t, *J* = 5.4Hz, 1H), 7.58 (s, 1H), 7.38 (s, 1H), 7.23 (d, *J* = 8.2 Hz, 1H), 7.15 (s, 1H), 7.10 (s, 1H), 6.90 (d, *J* = 8.2 Hz, 1H), 3.90 (s, 3H), 3.88 (s, 3H), 3.79 (dd, *J* = 13.5, 6.7 Hz, 2H), 3.04 (t, *J* = 7.5 Hz, 2H), 2.35 (s, 3H); ^13^C NMR (101 MHz, DMSO–*d*_6_) δ 158.70, 154.14, 148.70, 146.49, 135.13, 128.07, 127.01, 123.19, 122.95, 118.54, 112.04, 111.54, 109.07, 107.54, 102.47, 99.99, 56.47, 56.12, 42.00, 25.38, 21.74; HRMS (ESI) m/z calcd C_21_H_22_N_4_O_2_ [M+H]^+^ 363.1821, found 363.1816.

### N-(2-(5-bromo-1H-indol-3-yl)ethyl)-6,7-dimethoxyquinazolin-4-amine (11h)

White solid; purity: 97%; yield: 72%; ^1^H NMR (400 MHz, DMSO–*d*_6_) δ 11.04 (br, 1H), 8.40 (s, 1H), 8.13 (t, *J* = 5.6 Hz, 1H), 7.82 (d, *J* = 1.7 Hz, 1H), 7.57 (s, 1H), 7.32 (d, *J* = 8.6 Hz, 1H), 7.27 (d, *J* = 2.1 Hz, 1H), 7.18 (dd, *J* = 8.6, 1.9 Hz, 1H), 7.10 (s, 1H), 3.90 (d, *J* = 6.6 Hz, 6H), 3.78 (dd, *J* = 13.5, 6.8 Hz, 2H), 3.05 (t, *J* = 7.3 Hz, 2H); ^13^C NMR (101 MHz, DMSO – *d*_6_) δ 158.85, 154.49, 153.24, 149.00, 144.50, 135.41, 129.75, 125.00, 123.79, 121.34, 113.87, 112.42, 111.43, 108.78, 106.27, 102.90, 56.67, 56.23, 42.06, 25.07; HRMS (ESI) m/z calcd C_20_H_20_BrN_4_O_2_ [M+H]^+^ 427.0770, found 427.0759.

### N-(4-(1H-indol-2-yl)butan-2-yl)-6,7-dimethoxyquinazolin-4-amine (12)

White solid; purity: 97%; yield: 60%; ^1^H NMR (400 MHz, DMSO – *d*_6_) δ 10.71 (s, 1H), 8.32 (s, 1H), 7.67 (s, 1H), 7.58 (d, *J* = 7.6 Hz, 1H), 7.49 (d, *J* = 7.6 Hz, 1H), 7.32 (d, *J* = 8.0 Hz, 1H), 7.09 (t, *J* = 7.1 Hz, 2H), 7.04 (d, *J* = 7.7 Hz, 1H), 6.94 (t, *J* = 7.2 Hz, 1H), 4.58 – 4.48 (m, 1H), 3.90 (dd, *J* = 9.9, 4.6 Hz, 6H), 2.77 (t, *J* = 6.6 Hz, 2H), 2.02 – 1.88 (m, 2H), 1.31 (d, *J* = 6.3 Hz, 3H); ^13^C NMR (101 MHz, CDCl_3_) δ 157.79, 154.22, 154.19, 148.79, 146.52 136.45, 127.22, 122.06, 121.39, 119.24, 118.75, 115.83, 111.19, 108.52, 107.79, 99.31, 56.22, 56.15, 47.07, 36.86, 22.15, 21.13; HRMS (ESI) m/z calcd C_22_H_24_N_4_O_2_ [M+H]^+^ 377.1978, found 377.1972.

### N-(2-(1H-benzo[d]imidazol-2-yl)ethyl)-6,7-dimethoxyquinazolin-4-amine (13)

White solid; purity: 98%; yield: 68%; ^1^H NMR (400 MHz, DMSO – *d*_6_) δ 9.31(br, 1H), 8.56 (s, 1H), 7.84 (s, 1H), 7.52 (dd, *J* = 5.9, 3.2 Hz, 2H), 7.19 (s, 2H), 7.18 (d, *J* = 3.2 Hz, 1H), 4.17–4.01 (m, 2H), 3.92 (s, 3H), 3.89 (s, 3H), 3.34–3.26 (m, 2H); ^13^C NMR (101 MHz, DMSO–*d*_6_) δ 159.33, 155.46, 152.92, 150.99, 150.93, 149.68, 149.66, 137.57, 137.46, 122.67 × 2, 114.82, 107.93, 103.66, 102.90, 56.97, 56.55, 31.18, 28.19; HRMS (ESI) m/z calcd C_19_H_19_N_5_O_2_ [M+H]^+^ 350.1617, found 350.1616.

### Protein expression and purification

The recombinant pET15b-PDE10A plasmid coding the catalytic domain (residues 446-789) was subcloned and purified according to the following protocols previously reported (Li et al., 2015). Then it was transferred into *E. coli strain* BL21 (Codonplus, Stratagene). The *E. coli* cells carrying the recombinant plasmid were cultured in an 2XYT medium (containing 100 μg/mL ampicillin and 30 μg/mL chloramphenicol) at 37°C until OD_600_ = 0.6-0.8. And then, 1 mM isopropyl-β-D-thiogalactopyranoside was added in to induce the PDE10A protein expression at 20°C for 24 h. The nickel nitriloacetic acid (Ni-NTA) column (Qiagen) was used for purifying PDE10A proteins. The concentration of the PDE10 fractions was estimated based on the absorbance at 280 nm (calculated by the ProtParam software). A typical batch of purification yielded 100-200 mg PDE10A protein from a 1.0 L cell culture.

### PDE10A enzymatic assays

The enzymatic activities of the catalytic domains of PDE10A were performed using ^3^H-cGMP solution in the assay buffer of 50 mM Tris pH = 7.5, 4 mM MgCl_2_, 1 mM DTT, and ^3^H-cGMP giving 20,000–30,000 cpm after the reaction terminated per assay. To a solution (DMSO) of test compounds in different concentration, the PDE10A enzyme in the assay buffer was added to perform the enzymatic reaction and then incubated at room temperature for 15 min. The assay was then terminated by addition of 0.2 M ZnSO_4_, Subsequently, 0.2 N Ba(OH)_2_ was added to precipitate the reaction product ^3^H-GMP, whereas unreacted ^3^H-cGMP remained in the supernatant. The radioactivity in the supernatant was measured in 2.5 mL Ultima Gold liquid scintillation cocktails (PerkinElmer) by a liquid scintillation counter (PerkinElmer 2910). The IC_50_ values of test compounds at PDE10A enzymes were measured by repeating of three independent experiments using the nonlinear regression method. Papaverine with an IC_50_ of 0.1 μM was used as the reference compound for enzymatic assay.

### Antioxidant assay

The modified oxygen radical absorbance capacity fluorescein (ORAC-FL) method was performed to determine the antioxidant activity (Ou et al., 2001; Dávalos et al., 2004). The reaction was diluted with 75 mM phosphate buffer (pH = 7.4), and the volume of the final reaction mixture was 200 μL in well. Test compound (20 μL) and fluorescein (120 μL, 150 nM final concentration) were placed in the well of a black 96 well optical bottom plates. After the mixture was incubated at 37°C for 15 min, AAPH solution (60 μL, 12 mM final concentration) was added rapidly. The plate was placed in a Spectrafluor Plus plate reader (Tecan, Crailsheim, Germany) and the fluorescence was recorded every minute for 4 h with an excitation wavelength at 485 nm and emission wavelength at 535 nM. Trolox was used as standard (1–8 μM, final concentration). A blank (fluorescein + AAPH) with phosphate buffer instead of test compounds and trolox calibration were performed for the assays of antioxidants. The samples were measured at different concentration (1–10 μM). All the reaction mixtures were prepared fourfold, and at least three independent assays were performed for each sample. Fluorescence in time course was normalized on basis of the blank (without antioxidants). The ORAC-FL values were calculated as the reported method. Final ORAC-FL values were expressed in μM of trolox equivalents. Ferulic acid was used as the positive reference compound, showing an ORAC-FL value of 1.6 trolox equivalents.

### Molecular docking studies

The starting conformation of synthesized compounds was generated using Accelrys Discovery Studio 2.5.5. The crystal structure of PDE10A protein with a bound inhibitor possessing the same quinazoline core (PDB code: 3QPN) was used as the reference (Helal et al., 2011). The binding site was defined by the co-crystallized PDE10A inhibitor in PDB entry 3QPN. The Surflex-dock in the software Tripos Sybyl 1.2 (Jain, 2003) was used to obtain the dominant docking conformations in this study.

## Results and discussion

### Rational design of PDE10 inhibitors

**Figure 1:**
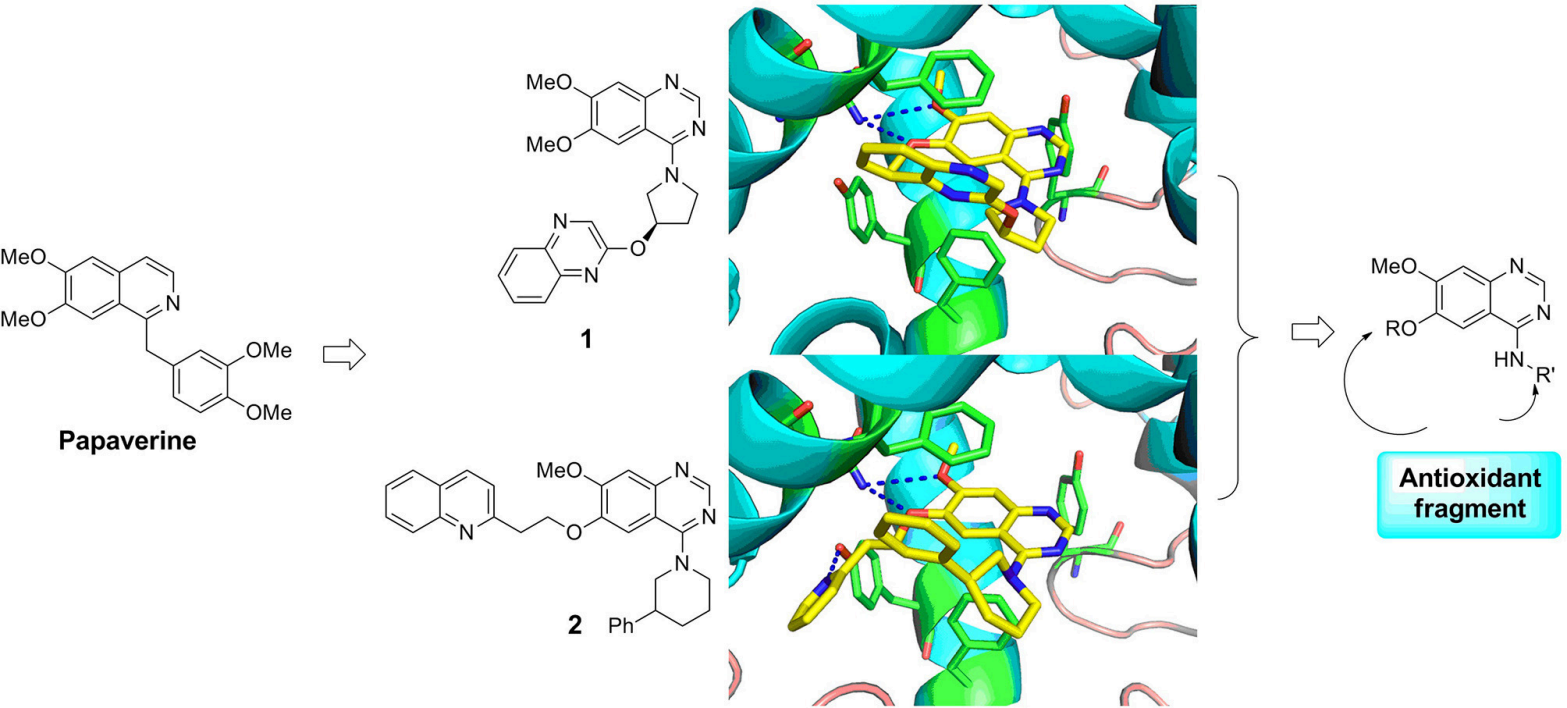
Rational design of novel PDE10 inhibitors with antioxidant activity.

Papaverine, a natural drug used for the prevention of vasospasm in the clinic, has been proved to have good inhibitory activity toward PDE10A (IC_50_ = 10–300 nM) (Siuciak et al., 2006a). Based on the structure of papaverine, several quinazoline compounds have been developed as PDE10A inhibitors such as compound **1** and compound **2** (Figure 1; Chappie et al., 2007; Helal et al., 2011). Observed from the crystal structure of **1** and PDE10A complex, following information for further structural modification were obtained. Firstly, the quinazoline ring located in a hydrophobic clamp comprised of Phe719 and Phe686 in the PDE10A protein, forming π-π interaction with Phe719. Secondly, the 6,7-dimethoxy group in the quinazoline ring formed a bidentate interaction with Gln716 in the pocket. As Gln716 has been regarded as a conserved amino acid residue in PDE10A, the interaction with Gln716 is the main reason for the high affinity of compounds with PDE10A protein. Thus, in our designed compounds, the quinazoline ring was kept as core. Last but not least, the piperazine ring of **1** located outside of the catalytic site in PDE10 protein, providing room for introducing a fragment with antioxidant activity. Furthermore, compound **2**, a PDE10 inhibitor developed from **1**, had a quinoline ring attached on the quinazoline core in order to completely fill the selectivity pocket mainly composed of Tyr683, Met703 and Gly715 in the PDE10A protein. Occupying this unique pocket in PDE10A may significantly enhance the selectivity of the inhibitors over other PDEs. In addition, 6-position at the quinazoline core also provides space and synthetic possibilities for conveniently introducing a fragment. Based on these evidences, the strategy combining PDE10A inhibitory activity and antioxidant activity might improve the druggability of hits. Different fragment from antioxidants (such as melatonin, ferulic acid and lipolic acid) were attached on the 6-position or 4-position of quinazoline ring to form the designed compounds and went into the following synthetic work.

### Chemistry

**Scheme 1:**
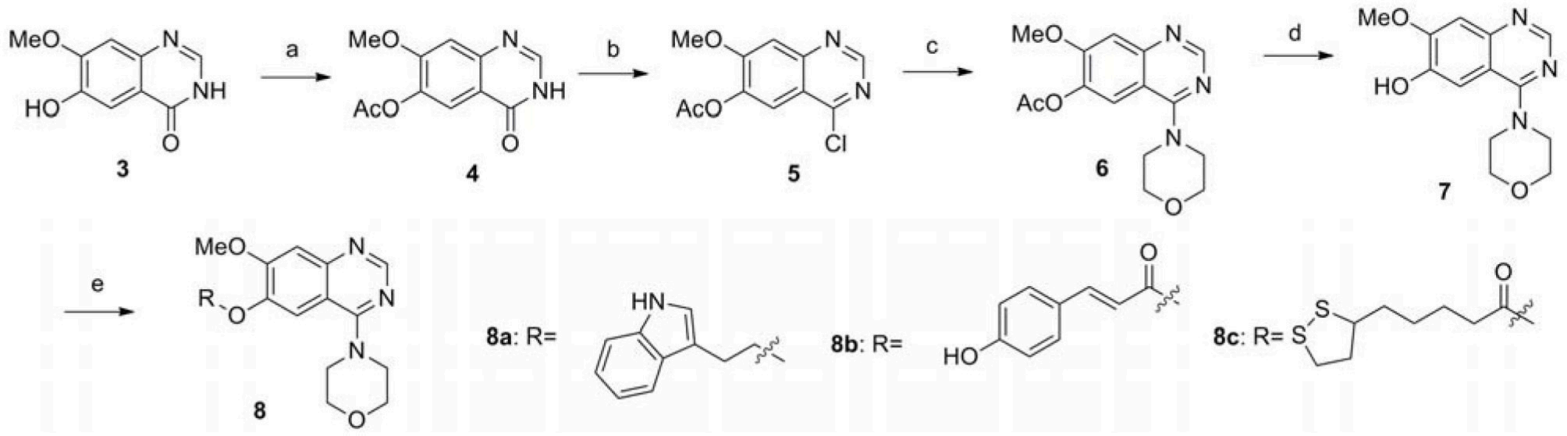
The synthesis route of compounds **8a**−**8c**. Reagents and conditions: (a) Aceticanhydride, pyridine, 100°C, 2 h; (b)SOCl_2_, DMF, 80°C, 2.5 h; (c) Morpholine, DMF, 80°C, 6 h; (d) Ammonia, methanol, reflux, 2 h; (e) The corresponding bromide, K_2_CO_3_, DMF, reflux for 3 h; or the corresponding acid, EDCI, DMAP, DMF, reflux overnight.

The synthetic route of compounds **8a-8c** is outlined in the Scheme 1 (Chandregowda et al., 2009). 6-hydroxy-7-methoxyquinazolin-4(3H)-one was reacted with acetic anhydride in pyridine to protect the phenolic hydroxyl group, providing the intermediated **4**. Chlorination of **4** was accomplished with SOCl_2_ to give 4-chloro-7-methoxyquinazolin-6-yl acetate **5**, which could be used in the reaction with the morpholine directly without further purification, providing 7-methoxy-4-morpholinoquinazolin-6-yl acetate **6**. Hydrolysis of the acetyl group of **6** was performed with concentrated ammonia in MeOH to give **7**. The final products **8a-8c** were obtained by the S_N_2 displacement with corresponding bromides or condensation reaction with corresponding acids in moderate yields.

**Scheme 2:**
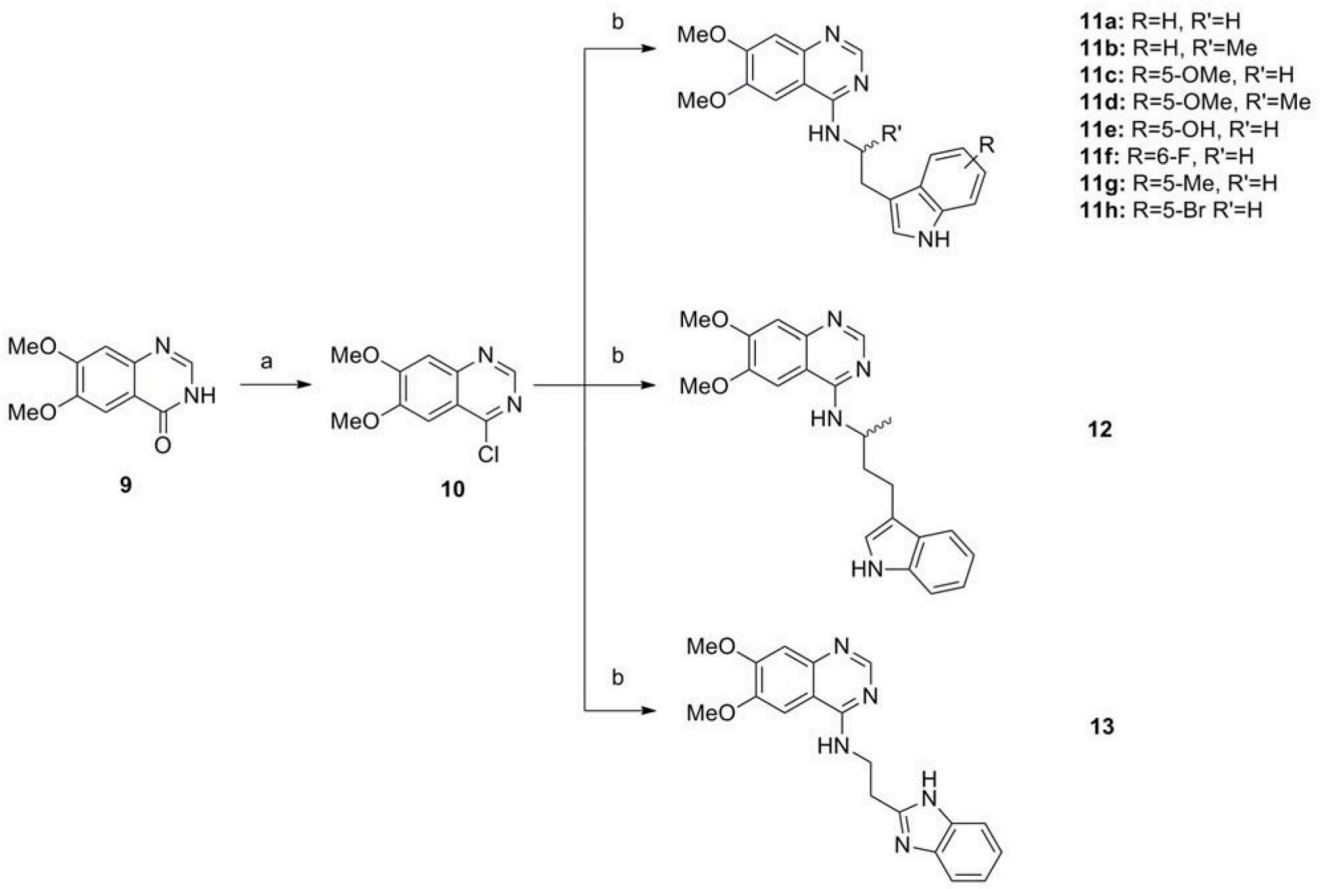
The synthesis route of compounds **11a**−**11h**, **12**, and **13**. Reagents and conditions: (a) SOCl_2_, DMF, 80°C, 2.5 h; (b) the corresponding amine, isopropanol, triethylamine, reflux.

The series of compound **11a-11h**, **12**, and **13** were synthesized in three steps (Scheme 2). With 6, 7-dimethoxyquinazolinone as the starting material, a following chlorination led to intermediate **10**, which reacted with different melatonin derivatives to acquire compounds **11a-11h**, **12**, and **13** in high yields (Garofalo et al., 2012; Min et al., 2016).

### Structure-activity relationships

Two series of compounds with antioxidant fragments on 4- or 6-position at the quinazoline core were synthesized. The inhibitory activities of these compounds toward PDE10A were evaluated with papaverine as the positive control. LogP (Octanol-Water Partition Coefficient) were calculated as the measure of lipophilicity, mostly drug candidates have LogP value below 5 based on Lipinski's Rule of Five. For our study, all synthetic and designed compounds have good LogP value. TPSA (Topological Polar Surface Area) provided a good estimate of proportion of compounds passed through BBB (blood-brain barrier), high penetration is necessary for drug candidates that targeted central nervous system (CNS) diseases. We were pleased to find that compound **8a**, with a fragment “2-(1H-indol-3-yl)ethyl” substituted on the 6-position, showed good inhibitory activity in the series of compounds **8a-8c**, although not as good as the papaverine and **1**, which also gave ORAC value of 1.0. Compound **8b** and **8c** only showed 26 and 24% inhibition on PDE10A at the concentration of 1 μM, respectively, despite that **8b** showed good TPSA value and antioxidant activity with ORAC value of 3.3 (Table 1). We concluded that the ester group in the linker caused steric hindrance in PDE10A pocket, resulting the low inhibitory activities.

**Table 1:** The Inhibitory activities against PDE10A, TPSA, LogP, and oxygen radical absorbance capacity of compounds **8a**-**8c**, **11a**-**11h**, **12**, and **13**.

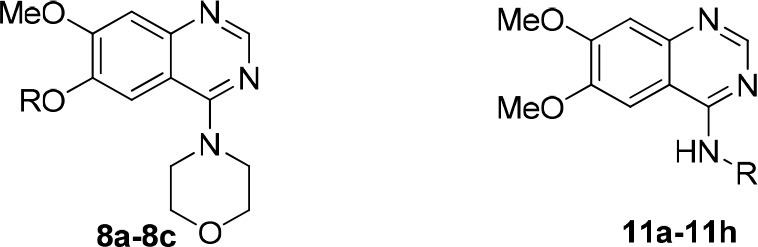						
**Compound**	**R**	**PDE10 inhibitory assay**		**TPSA**^b^	**LogP**^b^	**ORAC**^c^
		**Inhibition ratio (1** μ**M) (%)**	**IC**_50_ **(**μ**M)**^a^			
8a	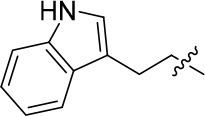	57	–	67.719	3.942	1.0 ± 0.1
8b	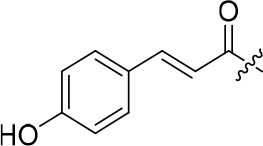	26	–	90.781	3.418	3.3 ± 0.1
8c	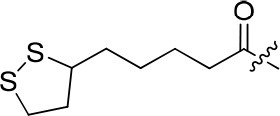	24	–	69.965	3.991	0.3 ± 0.01
11a	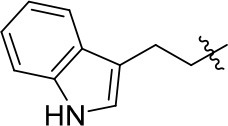	72	0.76 ± 0.09	68.247	3.901	1.3 ± 0.1
11b	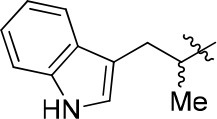	66	–	68.247	4.278	1.5 ± 0.1
11c	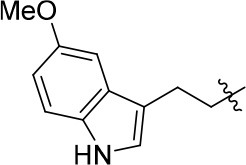	49	–	77.177	3.885	2.2 ± 0.03
11d	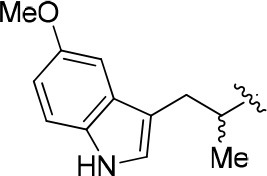	56	–	77.177	4.262	1.3 ± 0.1
11e	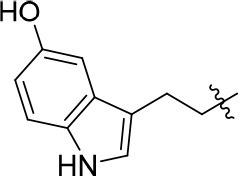	76	0.64 ± 0.05	89.063	3.659	2.3 ± 0.2
11f	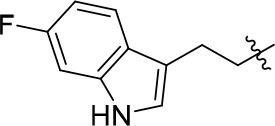	78	0.33 ± 0.04	68.247	4.106	1.0 ± 0.1
11g	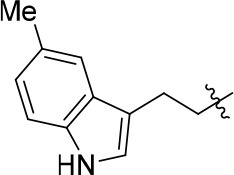	64	–	68.247	4.387	1.0 ± 0.2
11h	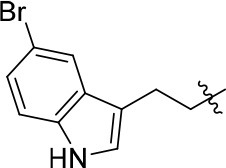	66	–	68.247	4.649	1.9 ± 0.1
12	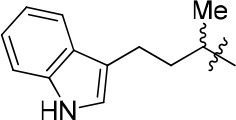	84	0.24 ± 0.02	68.247	4.735	1.3 ± 0.2
13	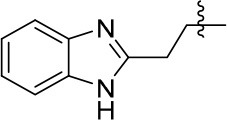	80	0.68 ± 0.09	79.5	3.243	0.15 ± 0.02
Papaverine	–	–	0.1	–	–	–
Ferulic acid	–	–	–	–	–	1.6 ± 0.1

a *IC_50_ values are given as the mean of three independent determinations*.

b *PSA and LogP values are calculated by Accelrys Discovery Studio 2.5.5*.

c *ORAC results are expressed as trolox equivalents*.

**Figure 2:**
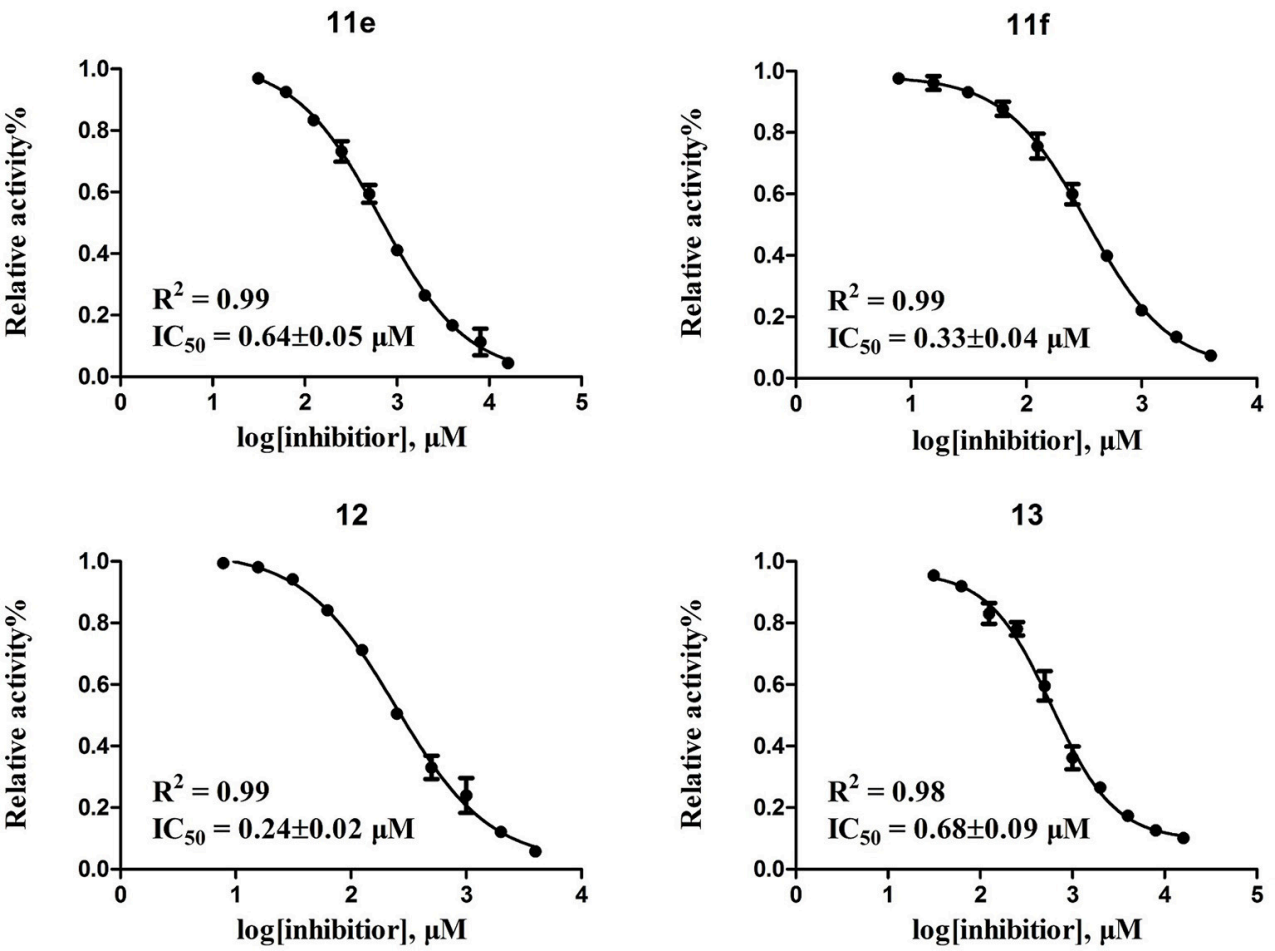
Inhibitory curves of four of the most potent compounds toward PDE10A.

According to the structures of reported PDE10/inhibitor complexes, Gln716 and Tyr683 in the PDE10 catalytic domain are two key amino residues for the interaction between inhibitors and PDE10 protein. From this point, we hypothesized that introducing a long chain at the 6-position of our hit compounds to form an interaction with the residue Tyr683 may be useful for the improvement of inhibitory activity toward PDE10. In the other series of compounds **11a-11h, 12 and 13**, different groups were placed on the 4-position of the quinazoline core and the methoxy group on the 6-position remain unchanged. The results were encouraging. Compound **11a**, **11e**, **11f**, **12**, and **13** showed good PDE10A inhibitory activities with IC_50_ below 1 μM. As depicted in Figure 2, compound **11f** and **12** exhibited the best PDE10A inhibitory activities with the IC_50_ of 0.33 and 0.24 μM. However, they showed moderate antioxidant activities with ORAC (oxygen radical absorbance capacity) 1.0 and 1.3. Compound **11e**, giving the third best IC_50_ of 0.64 μM on PDE10A and good ORAC (oxygen radical absorbance capacity) value of 2.3, reached a compromise between the PDE10A inhibitory activity and antioxidant activity. From **11a** to **11b** and **12**, it can be observed that the inhibition on PDE10 was affected by introducing extra carbon atom and methyl group in the linker, while the antioxidant activity was not much affected. In contrast, the antioxidant activities were weakened from **11c** to **11d**, with the ORAC (oxygen radical absorbance capacity) of 2.2–1.3, we concluded that the slight change of introducing a methyl group may cause the steric hindrance (**11b**, **11d** and **12** compared to **11a**, **11c** and **11d**) and leading to impropriate occupation in PDE10 protein, thus responsible for the decreased inhibition on PDE10A. The predict TPSA value of **8b** and **11e** seems good for the BBB penetration. Taking all into consideration, compound **11e**, which showed good PDE10 inhibitory activity and antioxidant activity was good lead for the further modification.

### Molecular docking

**Figure 3:**
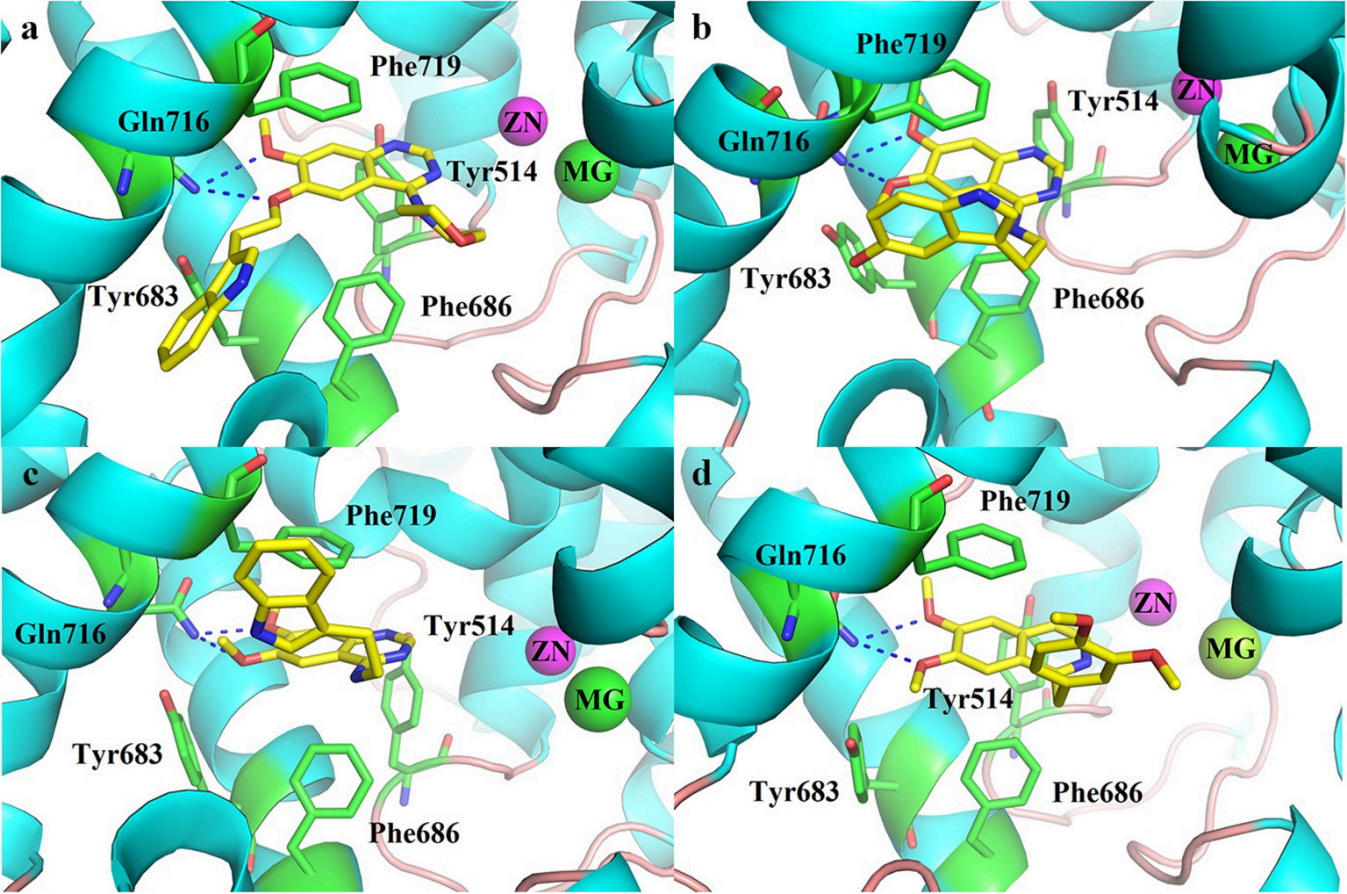
Docked conformation of **(a)** compound **8a**, **(b)** compound **11e**, **(c)** compound **11a** in complex with PDE10A and crystal structure of **(d)** papaverine in complex with PDE10A.

The molecular docking was performed to better understand the binding modes between the inhibitors and the PDE10 protein. The structure of PDE10 complex with papaverine has been reported in 2009 (PDB code: 2WEY), which clarified the interaction between papaverine and PDE10 (Andersen et al., 2009). All compounds of **8a**-**8c**, **11a-11h**, **12**, and **13** were docked into the PDE10 catalytic domain by the Surflex-dock in the software Tripos Sybyl 1.2 (Jain, 2003). From the docking results (Figure 3), we found that the quinazoline core of this series compounds occupied the same position as papaverine, and the oxygen of the quinazoline core formed a hydrogen bond interaction with the residue of Gln716. Besides, the quinazoline ring has the hydrophobic interactions with the residues of Phe719 and Phe686. These interactions have been reported to be the critical forces to determine the binding capacity of PDE10 inhibitors. In the series of compounds **8a**-**8c**, introducing groups at the 6-position of the quinazoline core could fill in the selective pocket of PDE10. However, no hydrogen bond was observed between compounds and Tyr683. The substituted groups of **8b** and **8c** might be too large in terms of volume size for the PDE10 selective pocket. Thus, the PDE10 inhibitory activities of them were weaker than that of **8a**. Compounds **11a**-**11h** had similar docking patterns since they structurally resemble each other. Besides, compound **8a** and **11a** have the same 2-(1H-indol-3-yl)ethyl group. By observing the docked conformations of **8a** and **11a** in complex with PDE10A, we found that the side chains stretch in different directions, respectively. The side chain of **8a** resides in the selective pocket of PDE10A, however, no hydrogen bond is formed with Tyr683. On the contrary, the side chain of **11a** stretches out of the catalytic site, possessing the same pattern as papaverine (Figure 3d).

## Conclusion

In summary, a series of novel PDE10A inhibitors with antioxidant activities were successfully designed and synthesized using a structure-based discovery strategy, which are potential pharmaceuticals as anti-neurodegenerative PD, AD, or ALS therapies. On the basis of the lead compound papaverine, 13 new quinazoline-based derivatives have been synthesized and evaluated by the inhibitory assays. Nine out of 13 compounds showed good PDE10A inhibitory activities at the concentration of 1 μM. Among these compounds, eight exhibited moderate to good antioxidant activity with the ORAC above 1.0. Especially worthy to mention is that compound **11e**, gave an IC_50_ value of 0.64 μM on PDE10A and good ORAC value of 2.3. In conclusion, this work has described a structure-based discovery strategy and the synthesized quinazoline-based derivatives with both PDE10A inhibitory activity and antioxidant activity, and might provide a new perspective for the development of novel PDE10A inhibitors.

## Author contribution

All authors listed have made a substantial, direct and intellectual contribution to the work, and approved it for publication.

### Conflict of interest statement

The authors declare that the research was conducted in the absence of any commercial or financial relationships that could be construed as a potential conflict of interest.

## Supplementary material

Presentation 1 ^1^H NMR and ^13^C NMR data for tested compounds.
