# Supplementary material for: Structure-Based Design, Synthesis, Biological Evaluation, and Molecular Docking of Novel PDE10 Inhibitors With Antioxidant Activities
Source: Front Chem. 2018 May 15;6:167. doi: 10.3389/fchem.2018.00167 (PMC5962708; doi:10.3389/fchem.2018.00167)
Supplement: Presentation 1 — 1H NMR and 13C NMR data for tested compounds. [file Presentation_1.PDF]

Supplementary Material

Structure-Based Design, Synthesis, Biological Evaluation, and Molecular Docking of Novel PDE10 Inhibitors with Antioxidant Activities

Jinxuan Li[#], Jing-Yi Chen[#], Ya-Lin Deng, **Qian Zhou, Yinuo Wu*, Deyan Wu*, and Hai-Bin Luo**

School of Pharmaceutical Sciences, Sun Yat-sen University, Guangzhou, 510006, China *****

*** Correspondence:**

Deyan Wu: e-mail, wudeyan3@mail.sysu.edu.cn
Yinuo Wu: e-mail, wyinuo3@mail.sysu.edu.cn

Contents

Page

1. NMR spectra of synthesized compounds...2

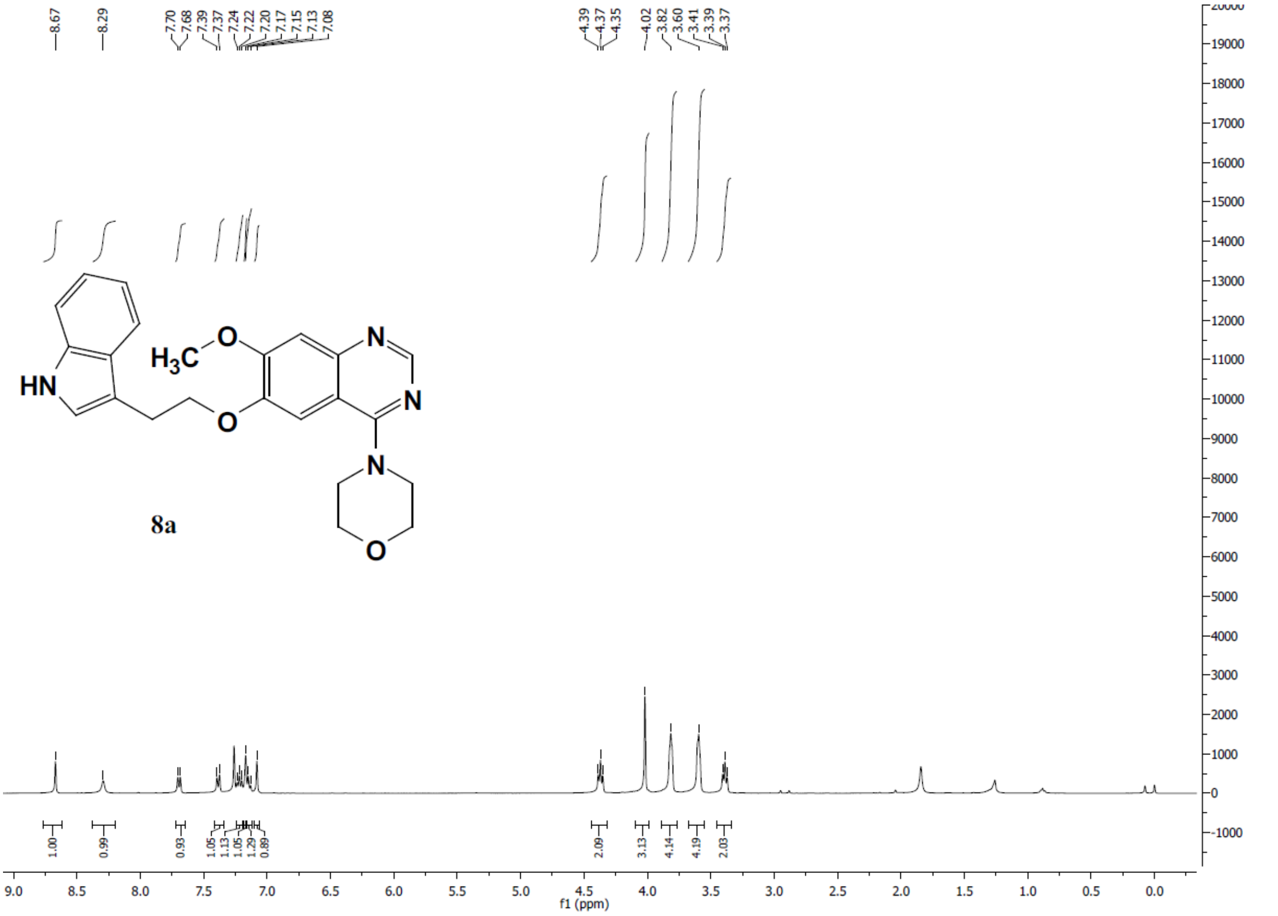
8.67
8.29
7.70 7.68 7.39 7.37 7.24 7.22 7.20 7.17 7.15 7.13 7.08
4.39 4.37 4.35
4.02 3.82 3.60 3.41 3.39 3.37
1.00 0.99 0.93 1.05 1.13 1.05 1.29 0.89 2.09 3.13 4.14 4.19 2.03
HN
H_3C
O
N
8a
f1 (ppm)

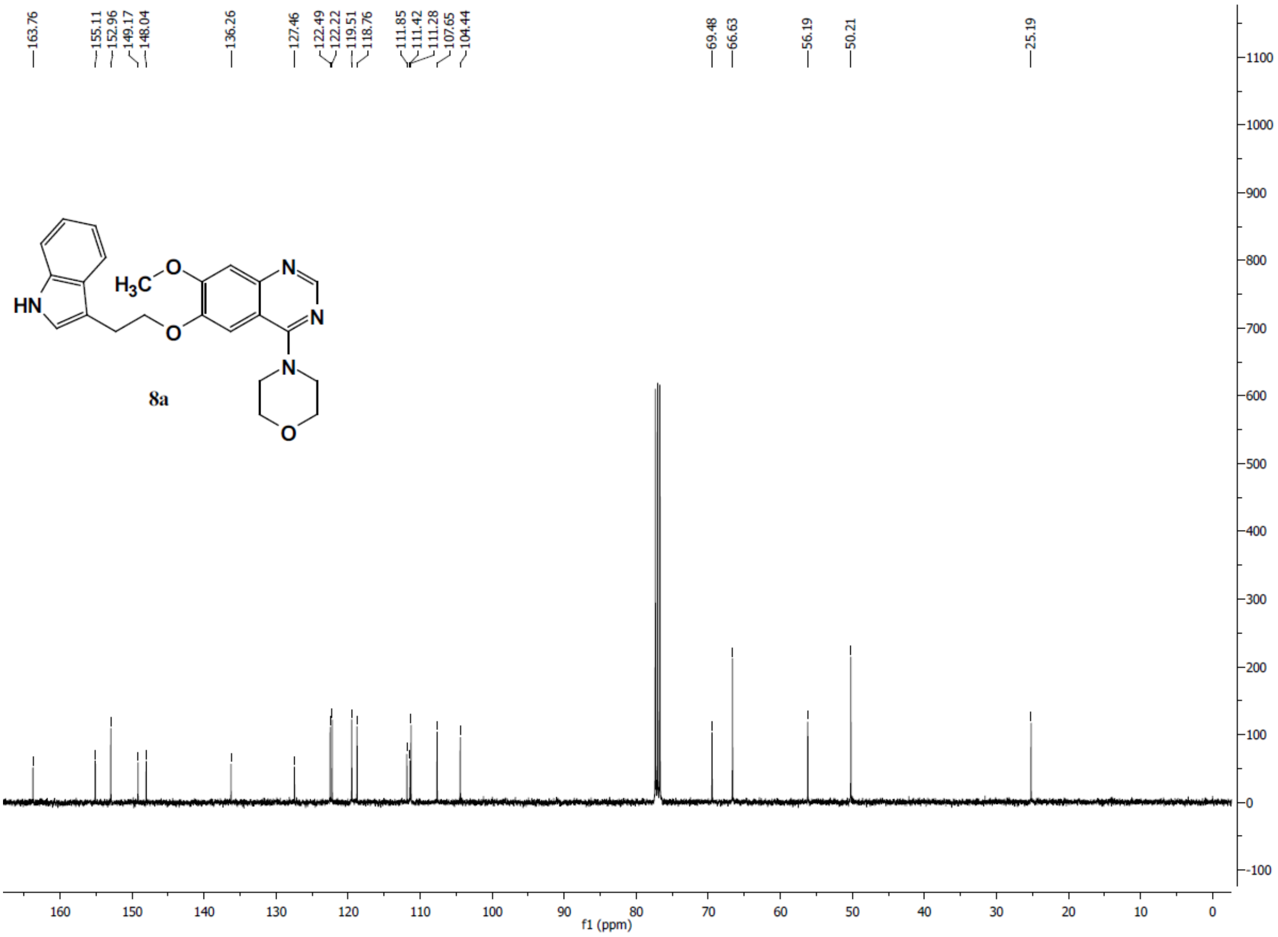
163.76
155.11 152.96 149.17 148.04
136.26
127.46 122.49 122.22 119.51 118.76
111.85 111.42 111.28 107.65 104.44
69.48 66.63
56.19
50.21
25.19
HN
H_3C
O
N
8a
f1 (ppm)

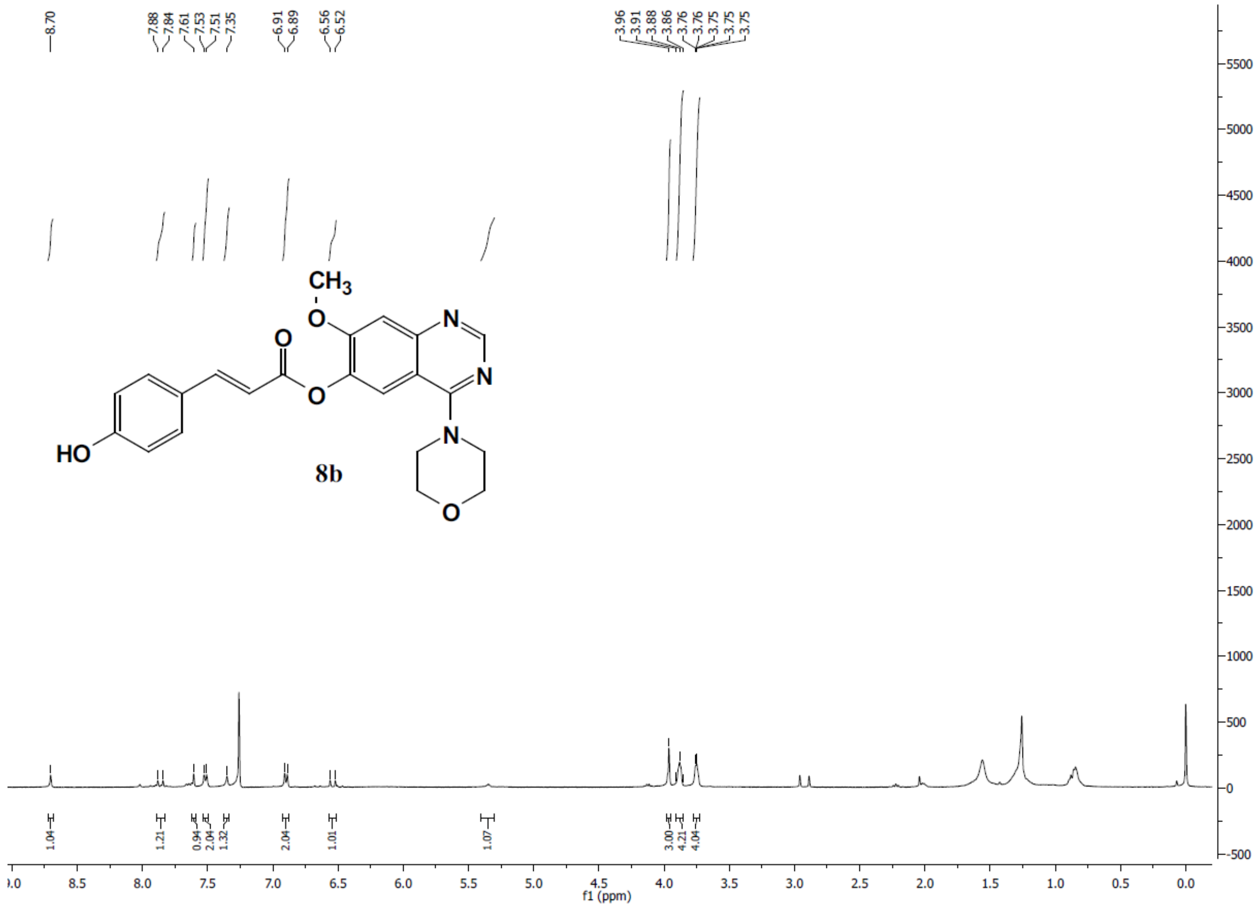
8.70
7.88 7.84 7.61 7.53 7.51 7.35
6.91 6.89
6.56 6.52
3.96 3.91 3.88 3.86 3.76 3.76 3.75 3.75 3.75
1.04 1.21 0.94 2.04 1.32 2.04 1.01 1.07 3.00 4.21 4.04
CH3
O
N
N
O
O
HO
N
O
8b
f1 (ppm)

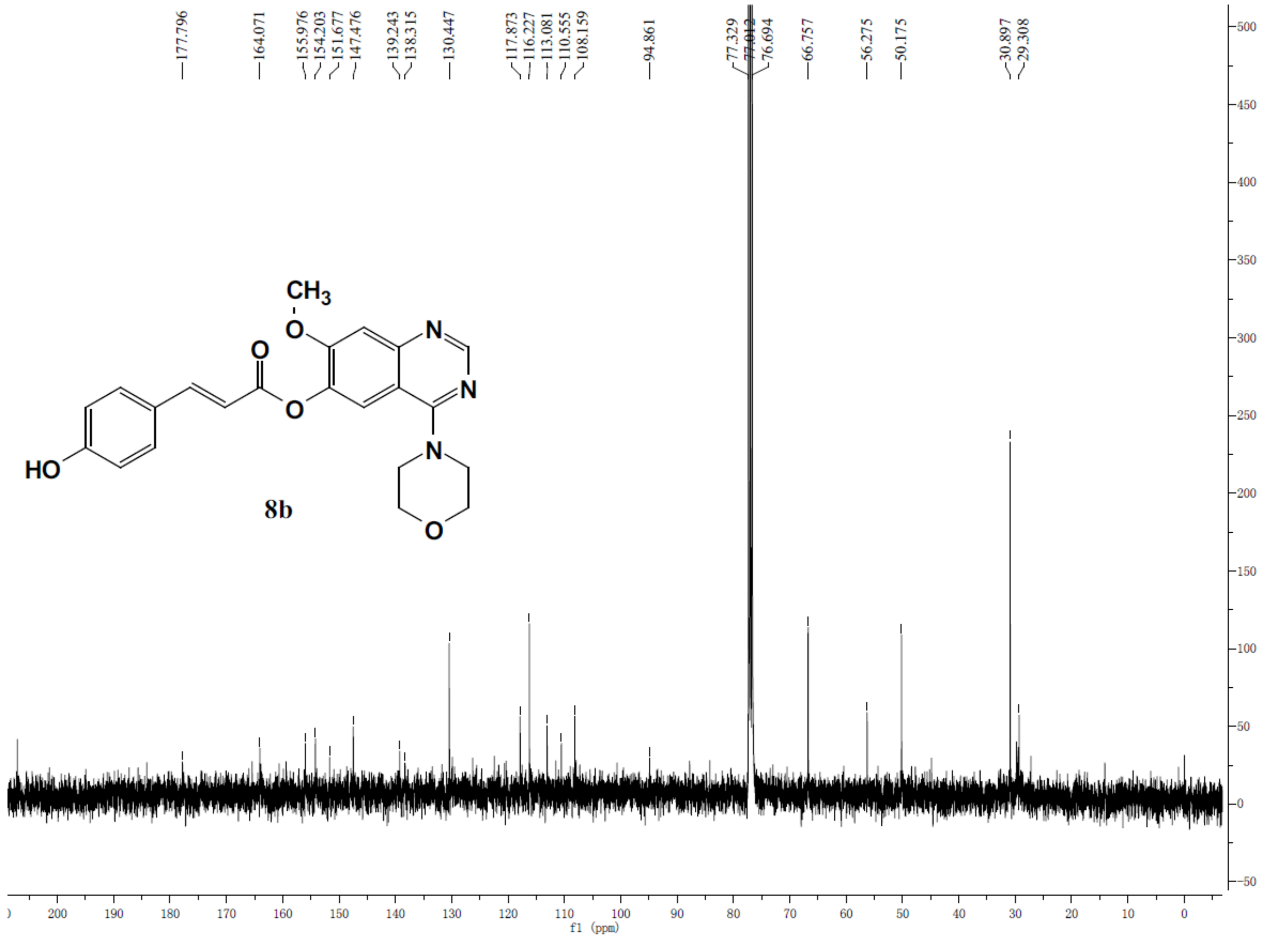
177.796
164.071
155.976 154.203 151.677 147.476
139.243 138.315
130.447
117.873 116.227 113.081 110.555 108.159
94.861
77.329 77.013 76.694
66.757
56.275
50.175
30.897 29.308
CH3
O
N
N
O
O
HO
N
O
8b
f1 (ppm)

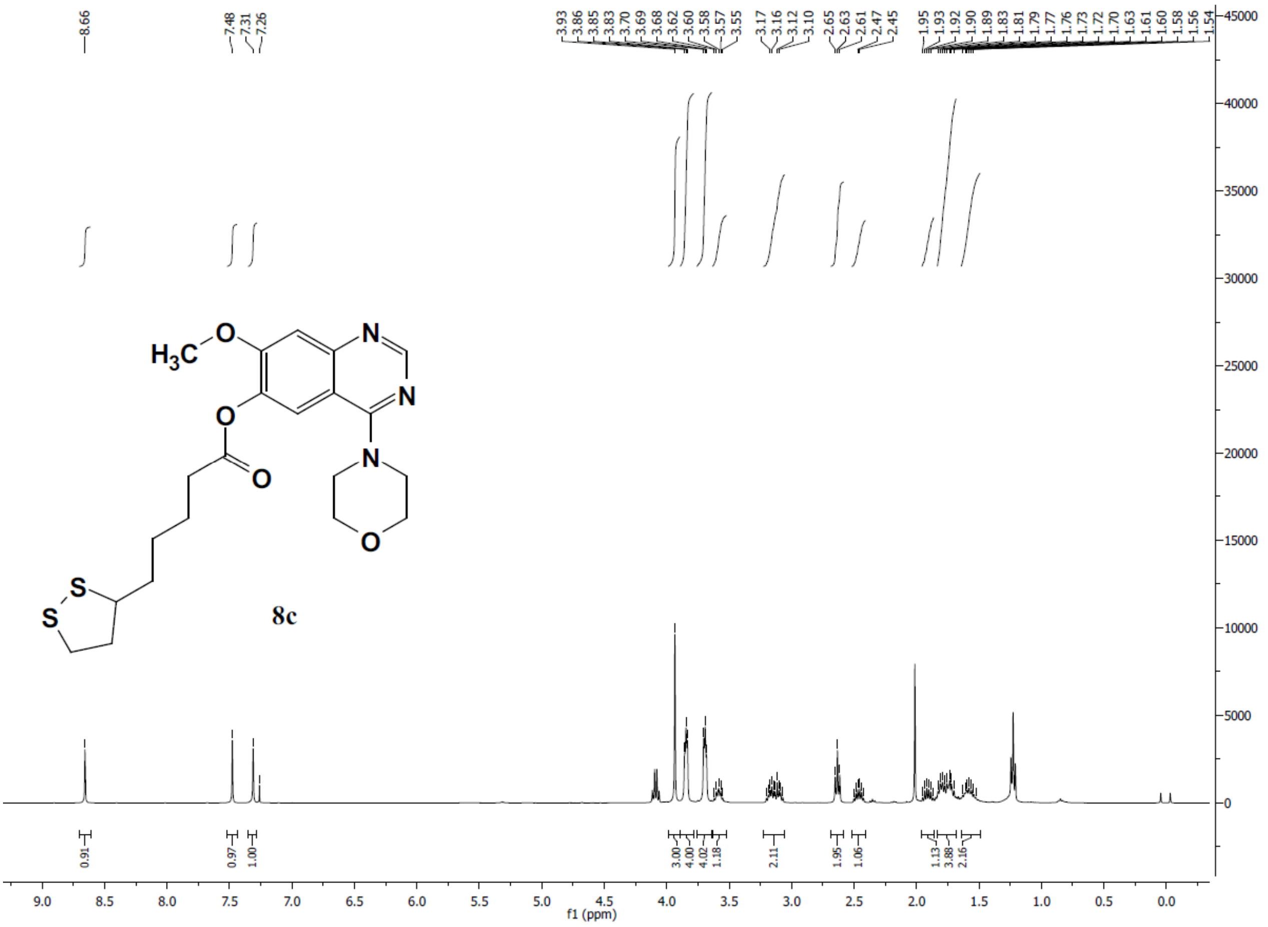
8.66
7.48
7.31
7.26
3.93 3.86 3.85 3.83 3.70 3.69 3.68 3.62 3.60 3.58 3.57 3.55
3.17 3.16 3.12 3.10
2.65 2.63 2.61 2.47 2.45
1.95 1.93 1.92 1.90 1.89 1.83 1.81 1.79 1.77 1.76 1.73 1.72 1.70 1.63 1.61 1.60 1.58 1.56 1.54
H3C
O
N
N
O
O
N
O
S
S
8c
0.91
0.97
1.00
3.00
4.00
4.02
1.18
2.11
1.95
1.06
1.13
3.88
2.16
f1 (ppm)

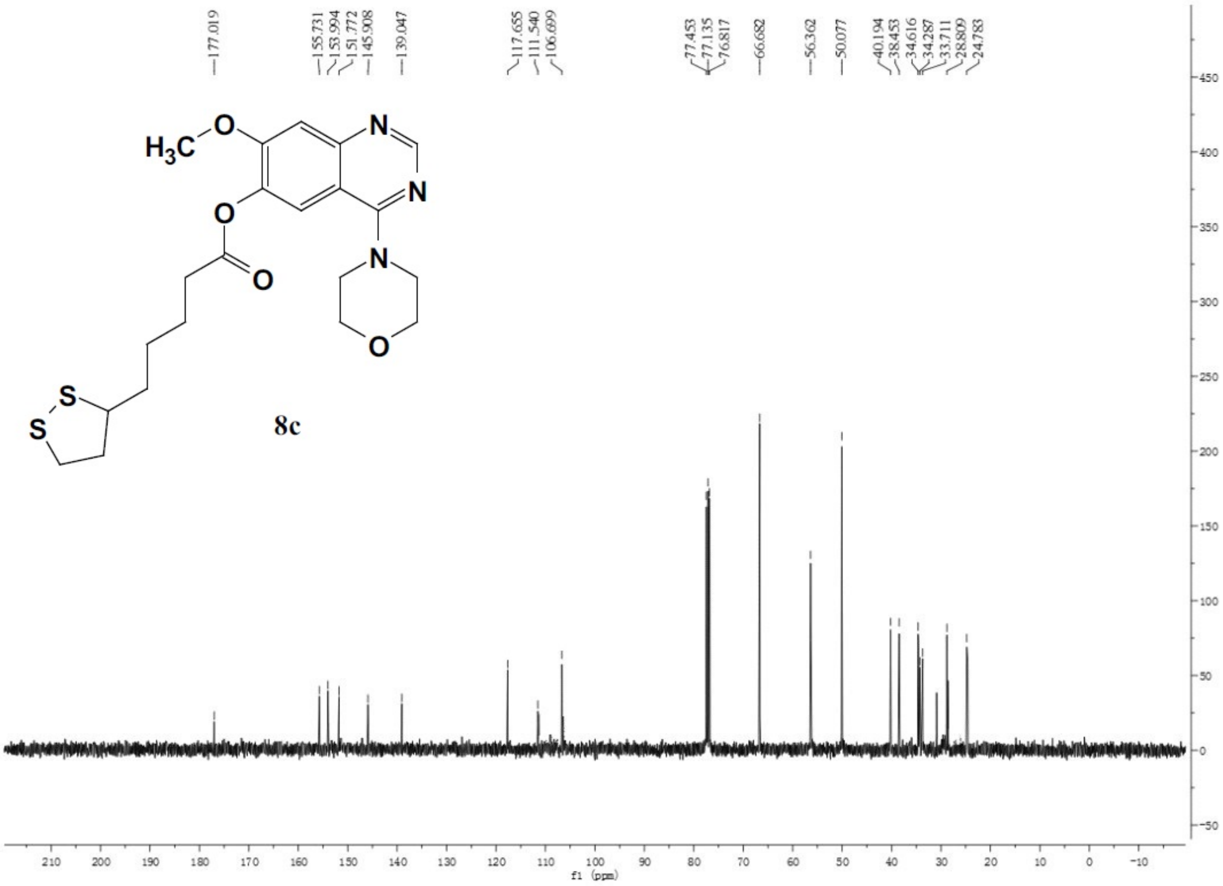
177.019
155.731
153.994
151.772
145.908
139.047
117.655
111.540
106.699
77.453
77.135
76.817
66.682
56.362
50.077
40.194
38.453
34.616
34.287
33.711
28.809
24.783
H3C
O
N
N
O
O
N
O
S
S
8c
f1 (ppm)

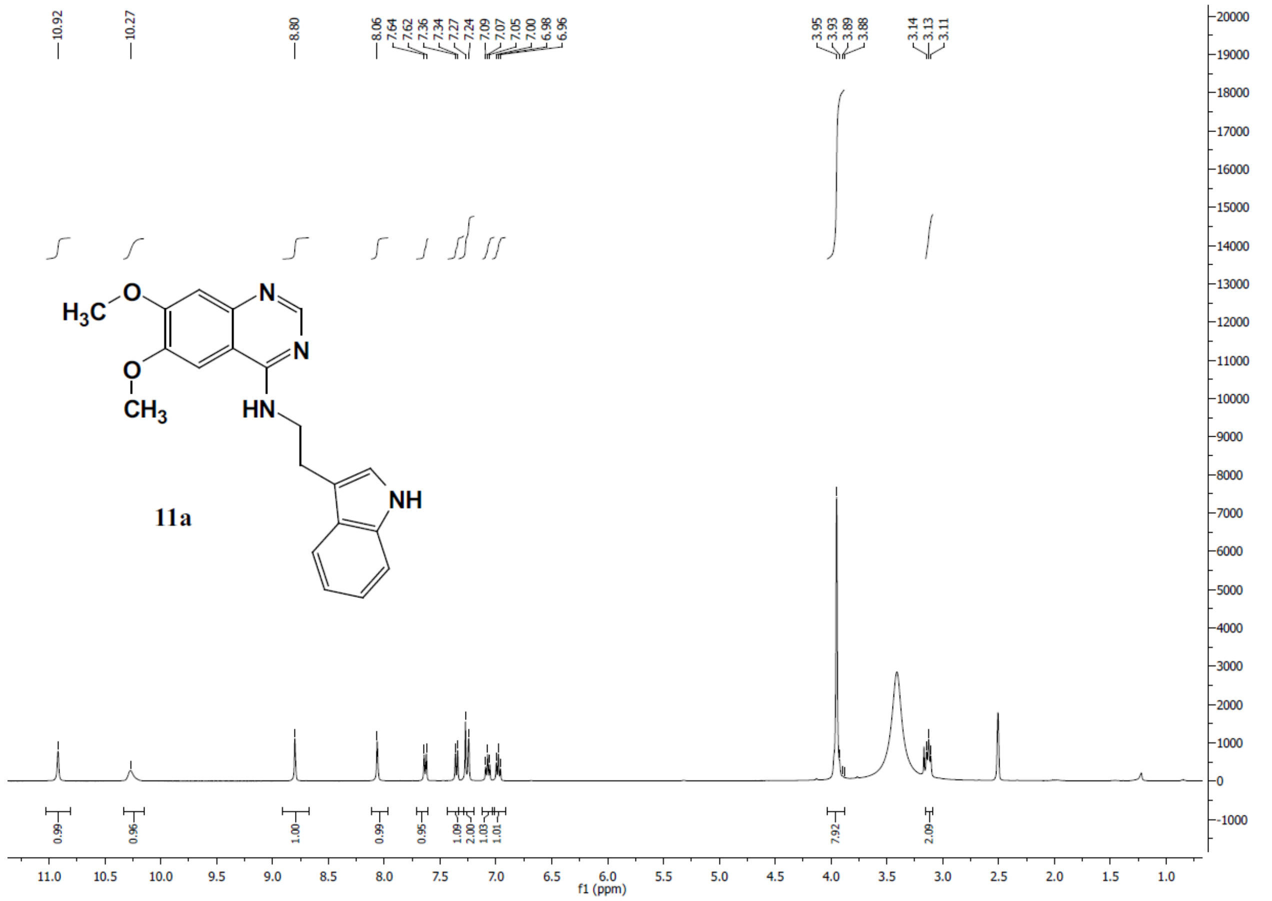
10.92
10.27
8.80
8.06 7.64 7.62 7.36 7.34 7.27 7.24 7.09 7.07 7.05 7.00 6.98 6.96
3.95 3.93 3.89 3.88
3.14 3.13 3.11
0.99 0.96 1.00 0.99 0.95 1.09 2.00 1.03 1.01 7.92 2.09
11a
f1 (ppm)

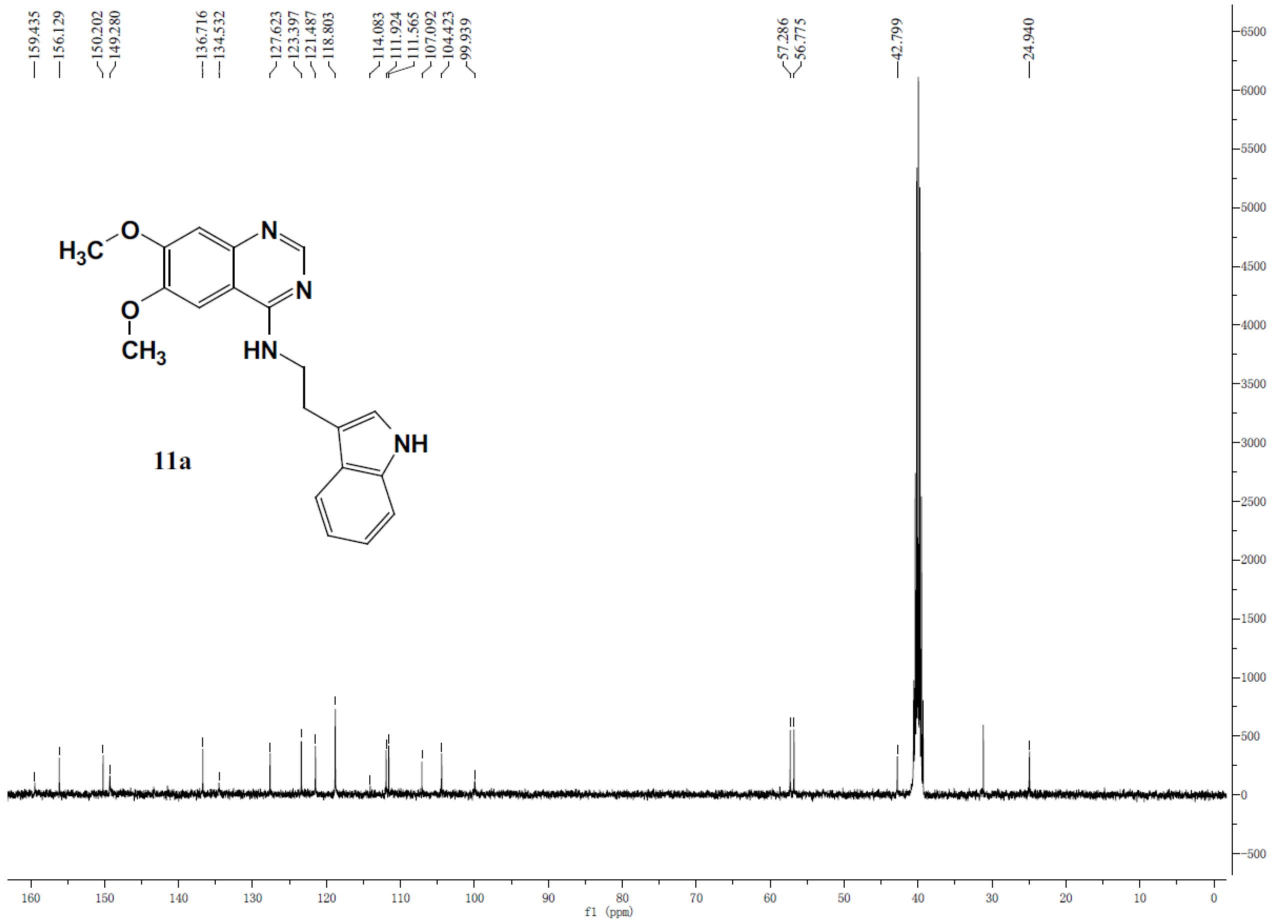
159.435 156.129 150.202 149.280
136.716 134.532
127.623 123.397 121.487 118.803
114.083 111.924 111.565 107.092 104.423 99.939
57.286 56.775
42.799
24.940
11a
f1 (ppm)

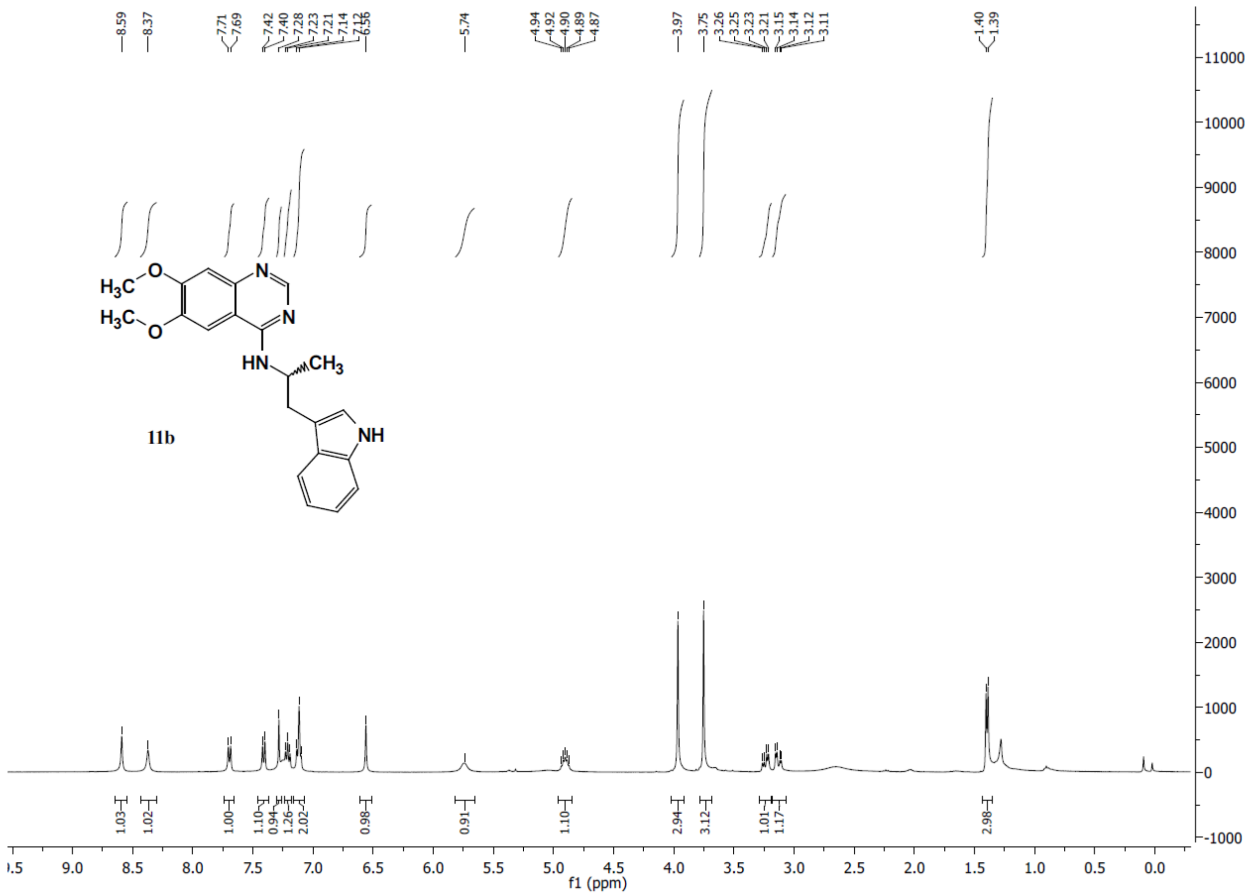

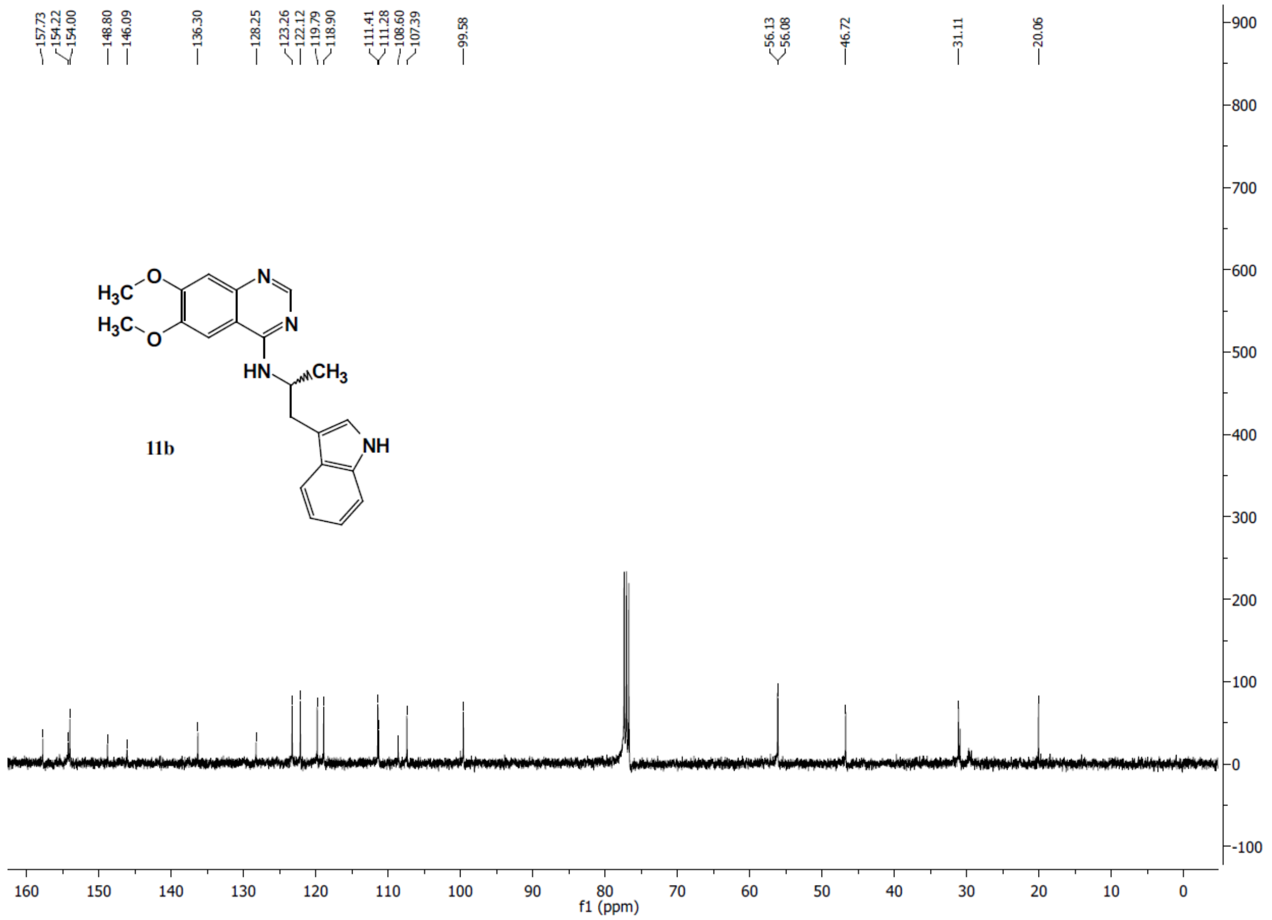

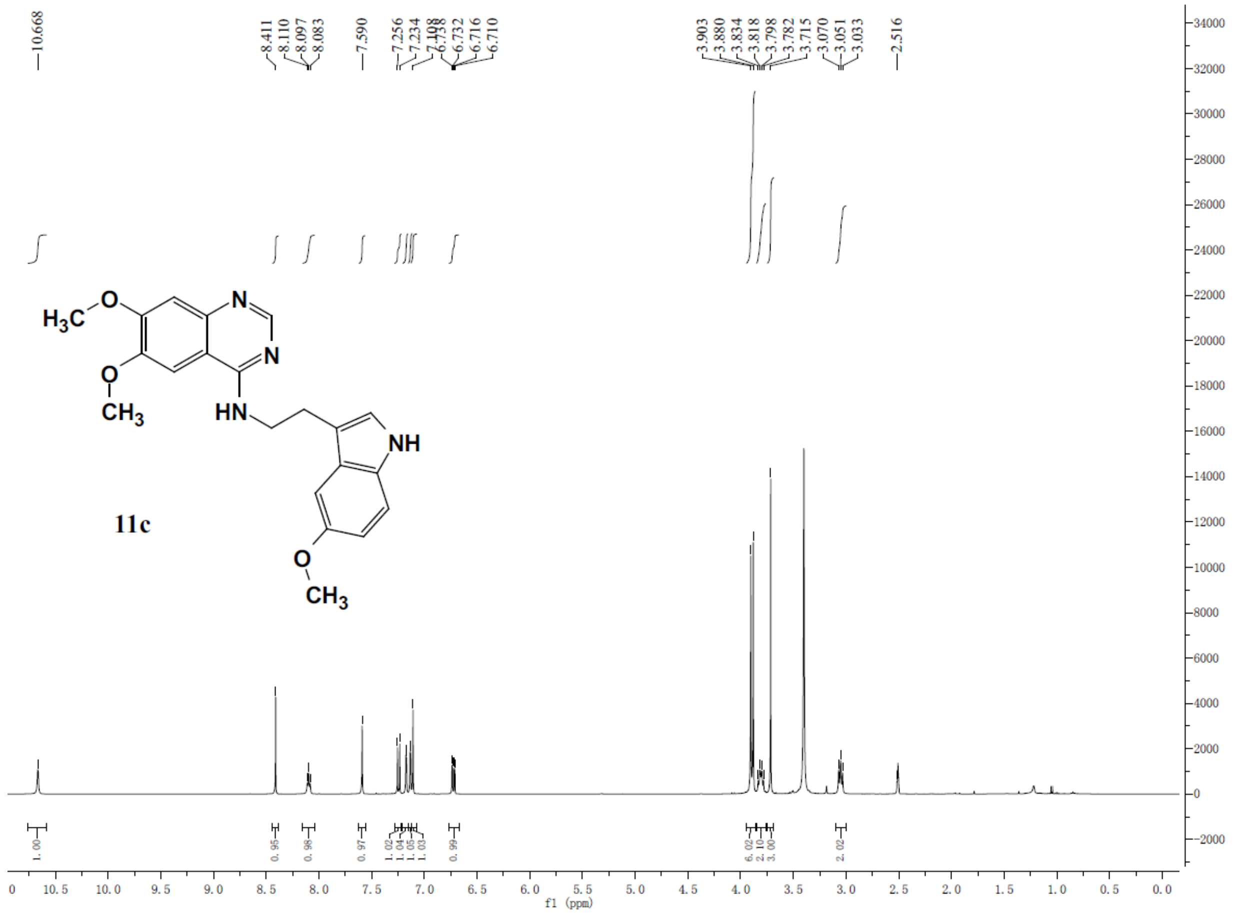
10.668
8.411
8.110
8.097
8.083
7.590
7.256
7.234
7.108
6.738
6.732
6.716
6.710
3.903
3.880
3.834
3.818
3.798
3.782
3.715
3.070
3.051
3.033
2.516
1.00
0.95
0.98
0.97
1.02
1.04
1.05
1.03
0.99
6.02
2.10
3.00
2.02
11c
f1 (ppm)

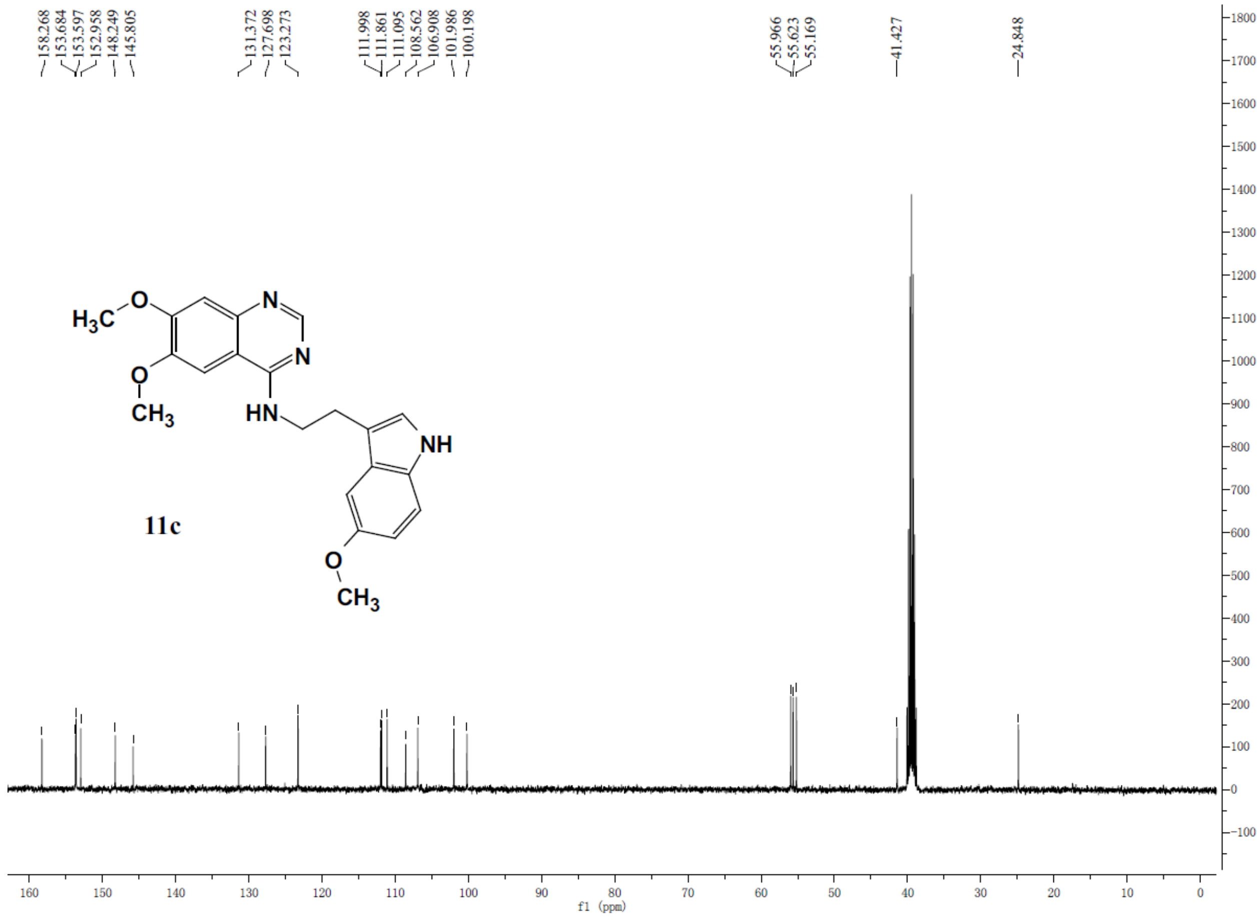
158.268
153.684
153.597
152.958
148.249
145.805
131.372
127.698
123.273
111.998
111.861
111.095
108.562
106.908
101.986
100.198
55.966
55.623
55.169
41.427
24.848
11c
f1 (ppm)

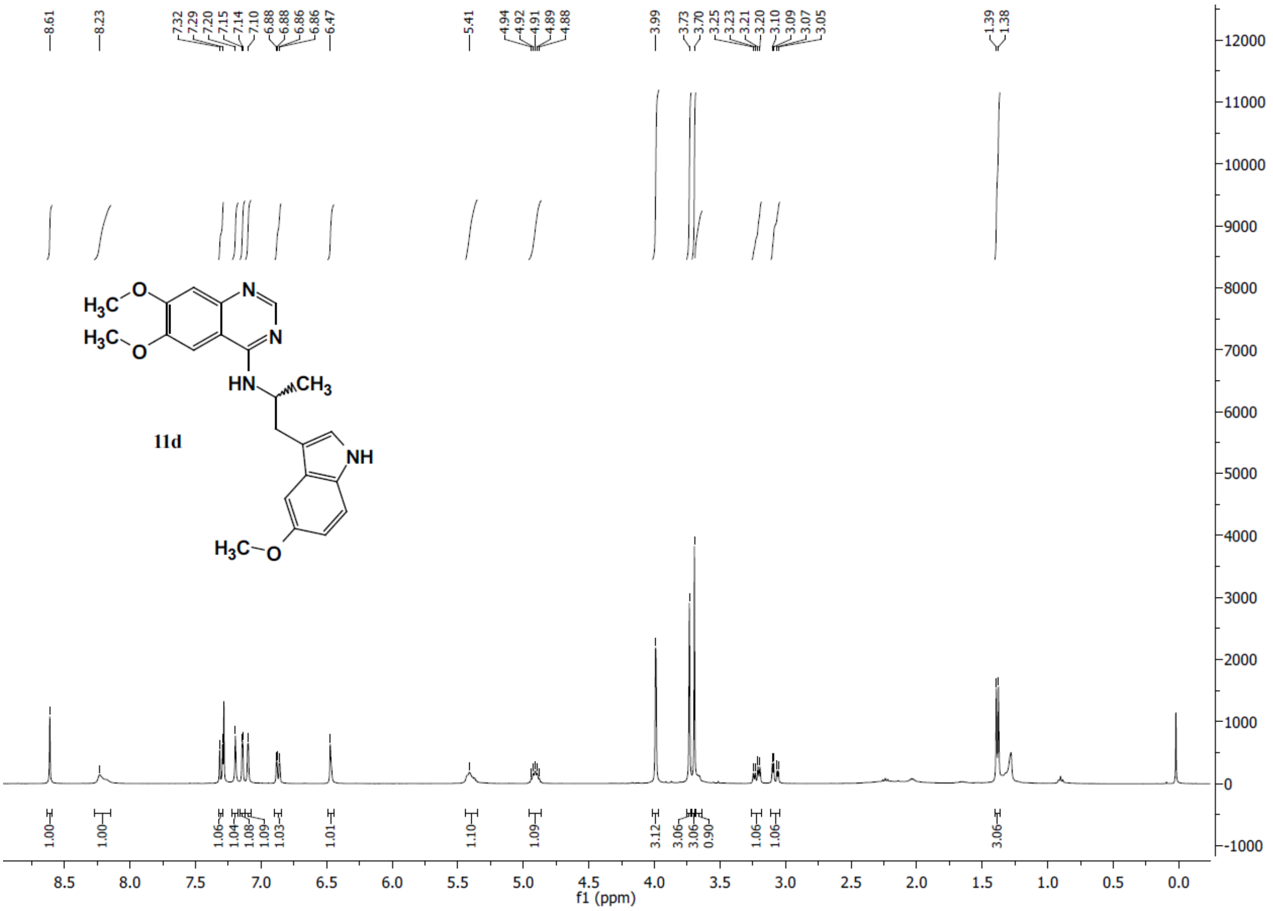
11d
f1 (ppm)

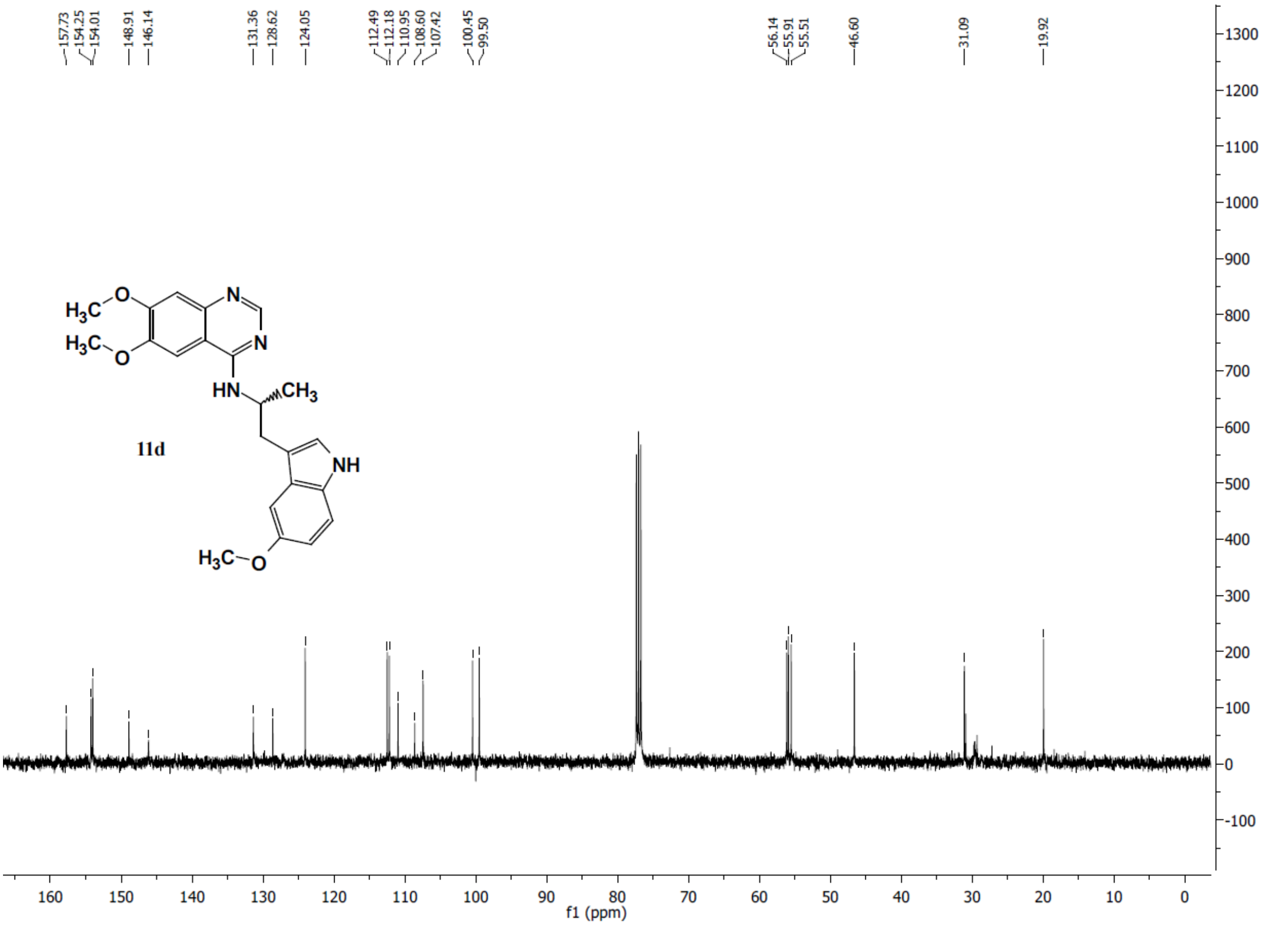
11d
f1 (ppm)

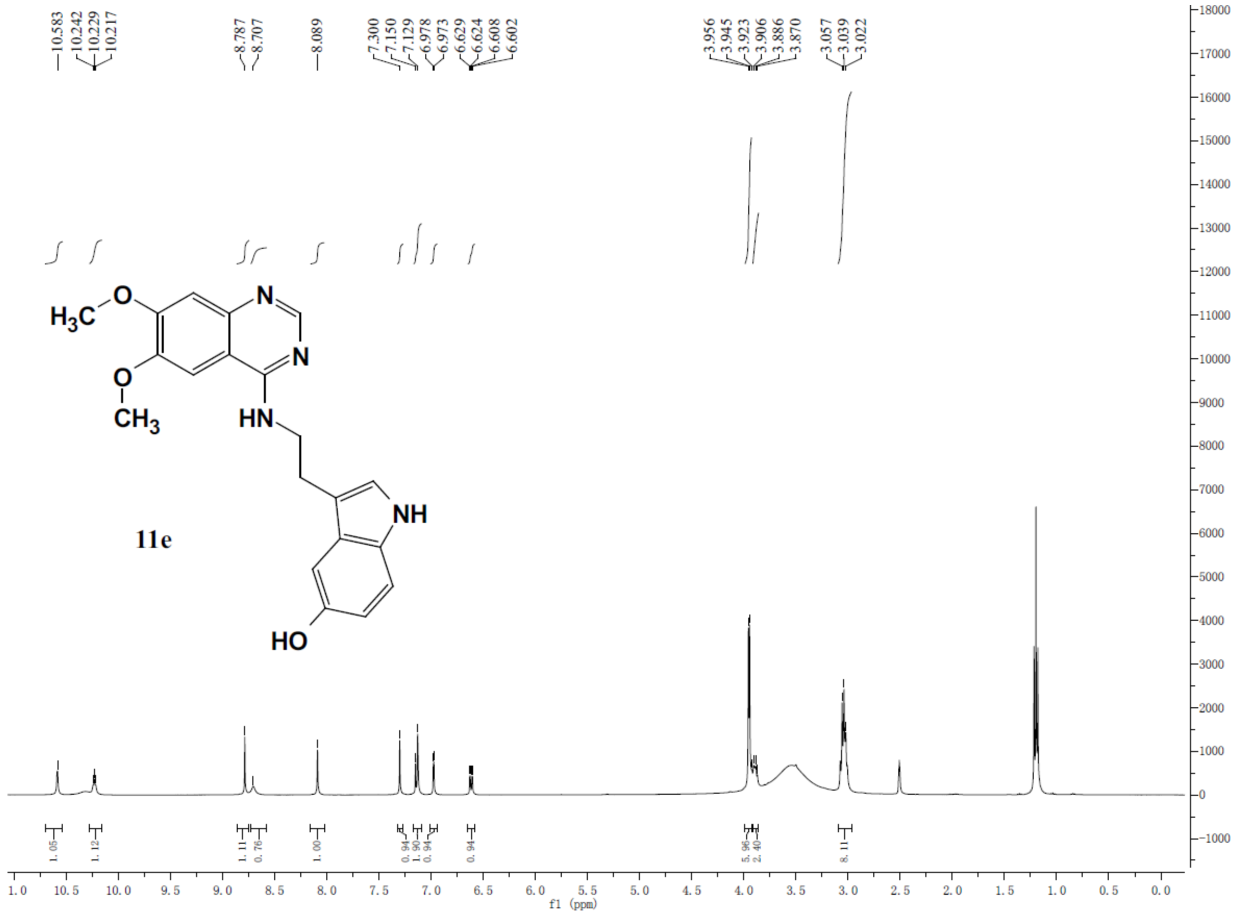
10.583
10.242
10.229
10.217
8.787
8.707
8.089
7.300
7.150
7.129
6.978
6.973
6.629
6.624
6.608
6.602
3.956
3.945
3.923
3.906
3.886
3.870
3.057
3.039
3.022
H_3C
O
N
N
O
CH_3
HN
NH
HO
11e
1.05
1.12
1.11
0.76
1.00
0.94
1.90
0.94
0.94
5.96
2.40
8.11
f1 (ppm)

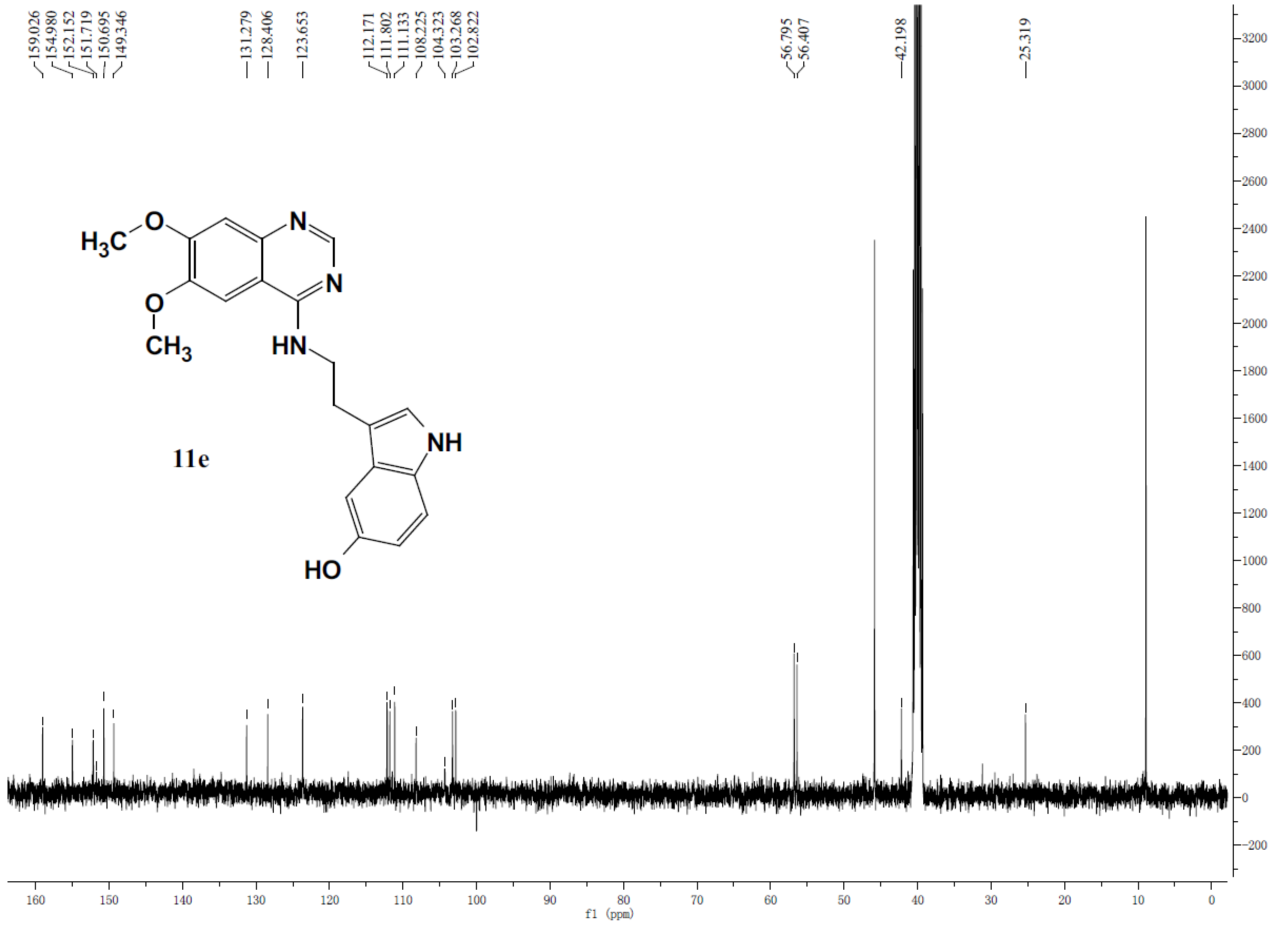
159.026
154.980
152.152
151.719
150.695
149.346
131.279
128.406
123.653
112.171
111.802
111.133
108.225
104.323
103.268
102.822
56.795
56.407
42.198
25.319
H_3C
O
N
N
O
CH_3
HN
NH
HO
11e
f1 (ppm)

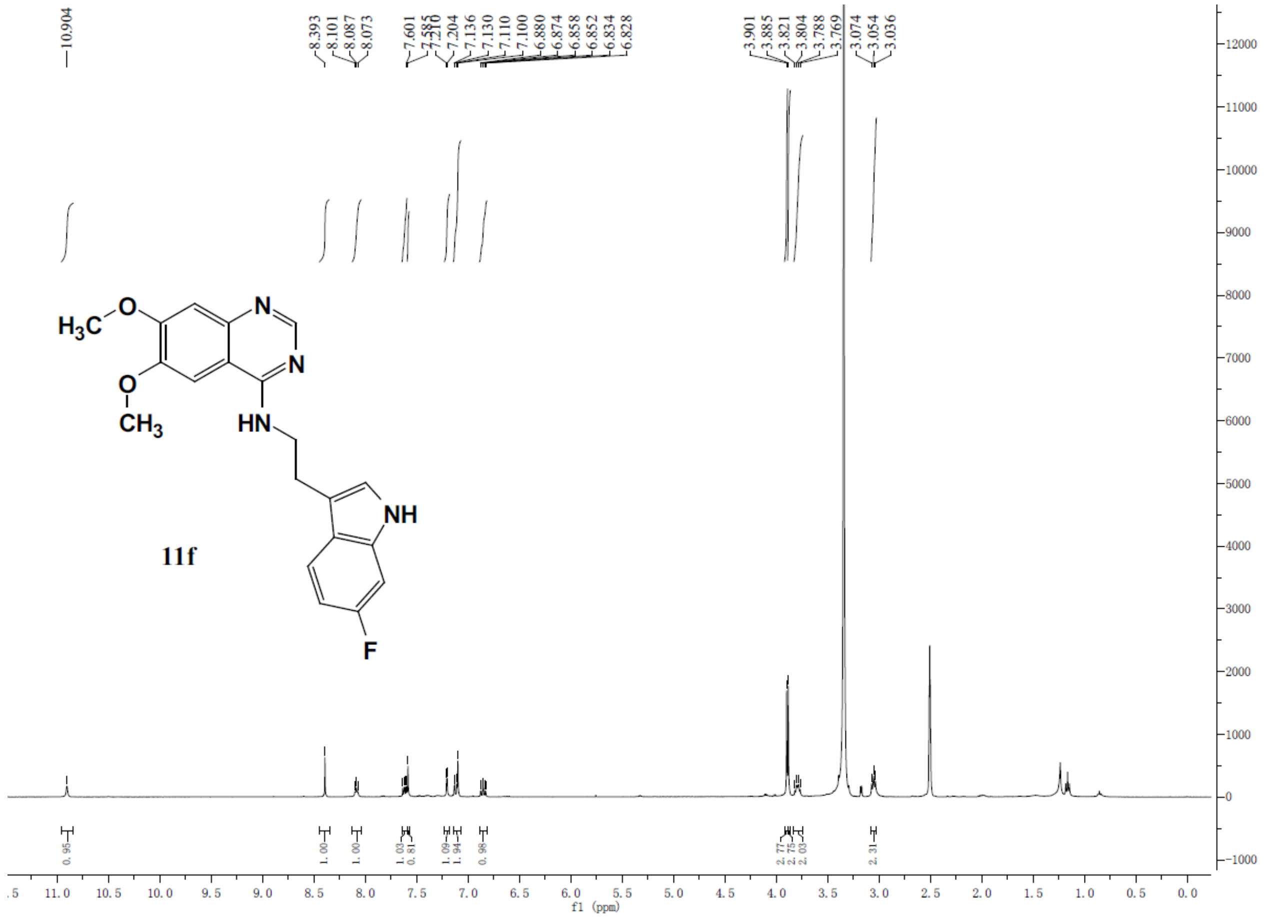

11f

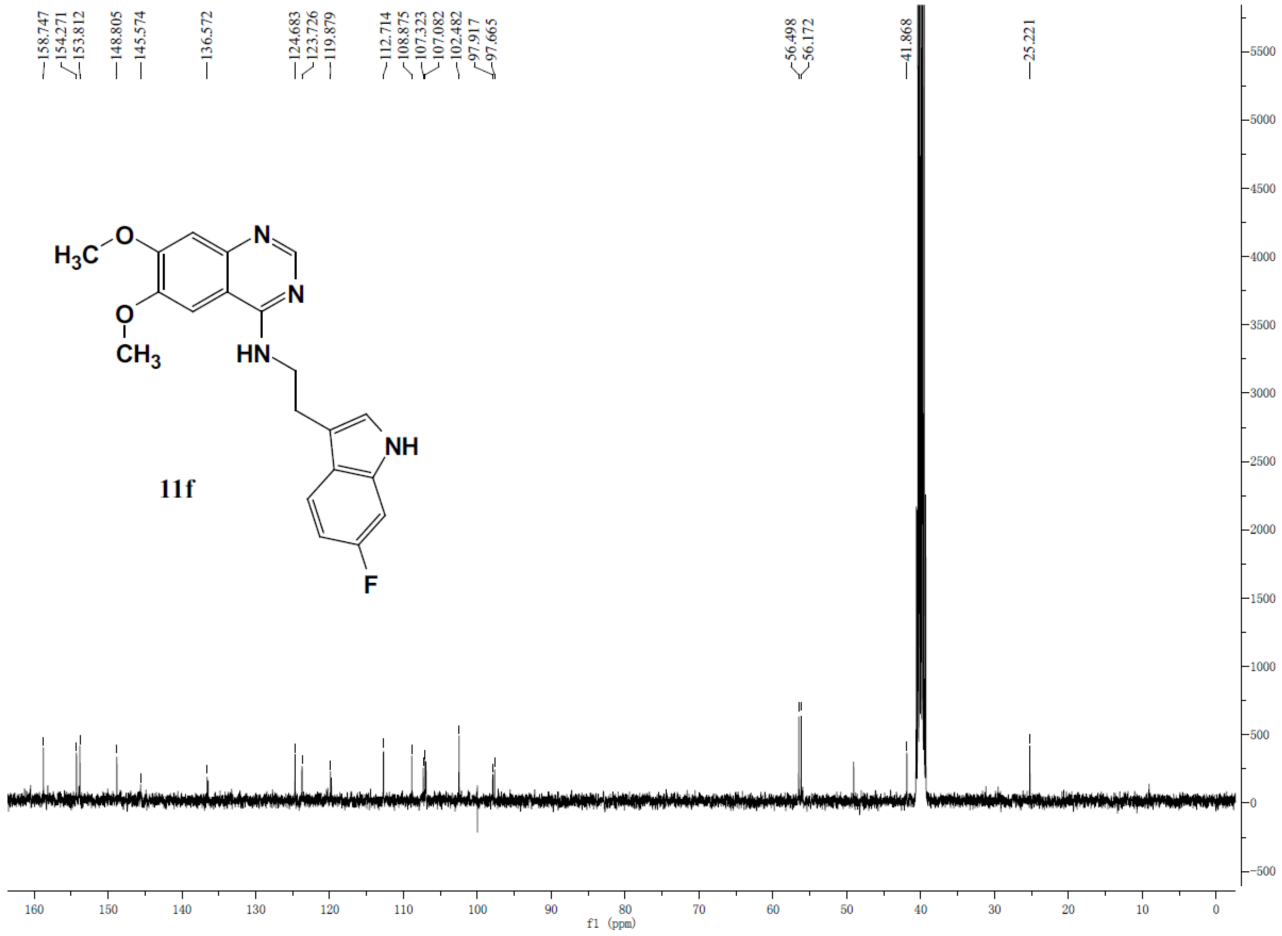

11f

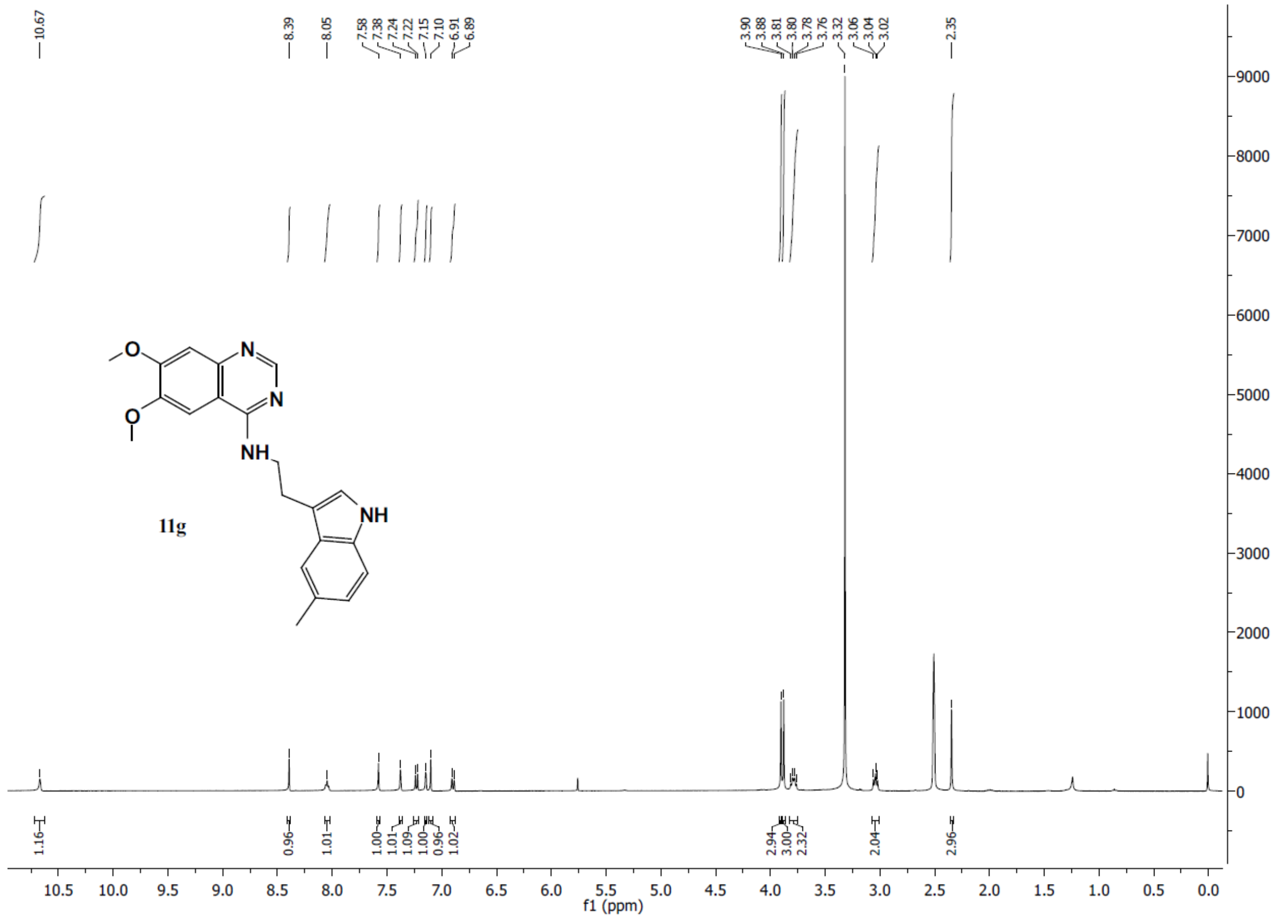
10.67
8.39
8.05
7.58
7.38
7.24
7.22
7.15
7.10
6.91
6.89
3.90
3.88
3.81
3.80
3.78
3.76
3.32
3.06
3.04
3.02
2.35
1.16
0.96
1.01
1.00
1.01
1.09
1.00
0.96
1.02
2.94
3.00
2.32
2.04
2.96
O
O
N
N
NH
NH
11g
f1 (ppm)

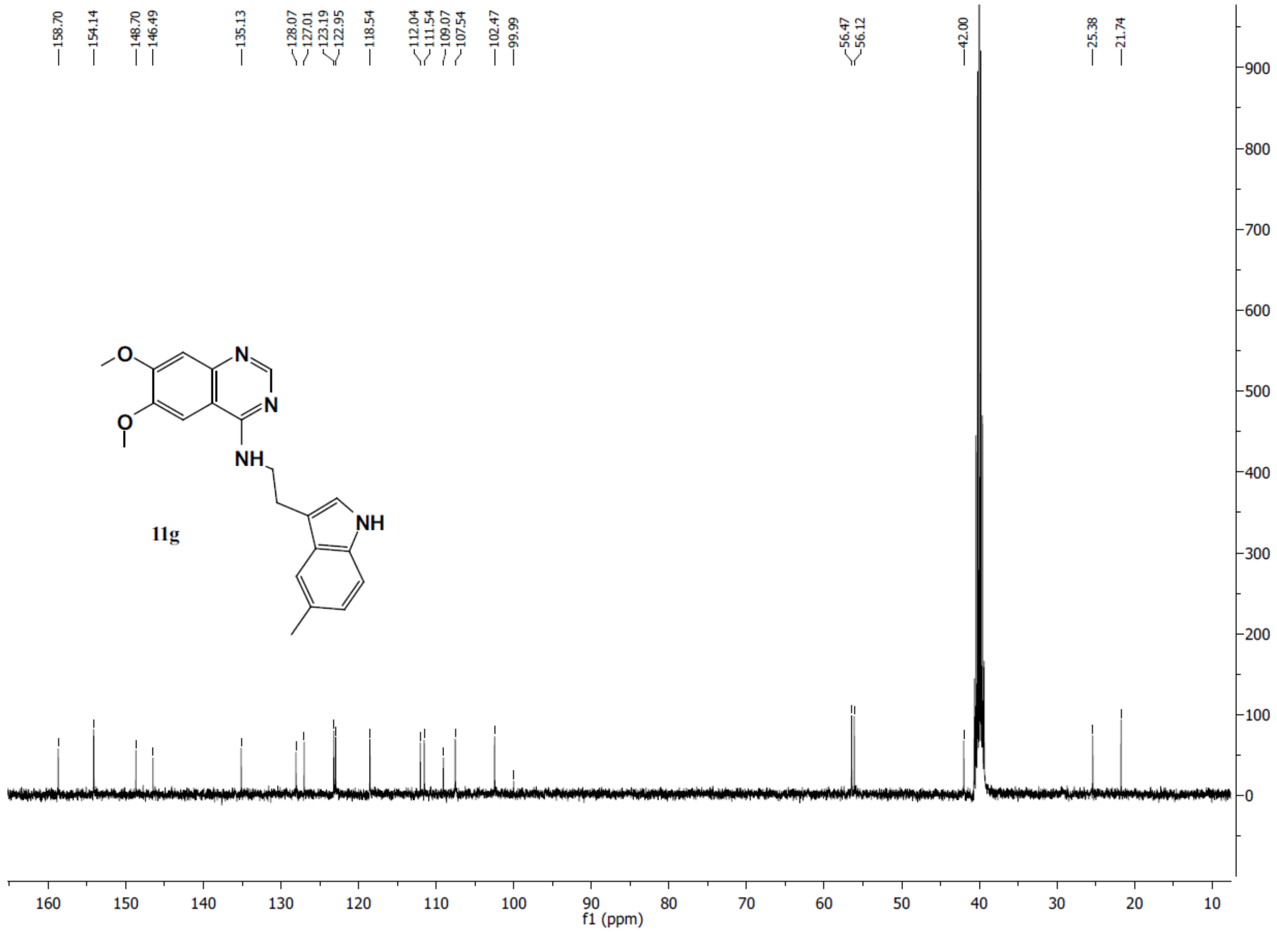
158.70
154.14
148.70
146.49
135.13
128.07
127.01
123.19
122.95
118.54
112.04
111.54
109.07
107.54
102.47
99.99
56.47
56.12
42.00
25.38
21.74
O
O
N
N
NH
NH
11g
f1 (ppm)

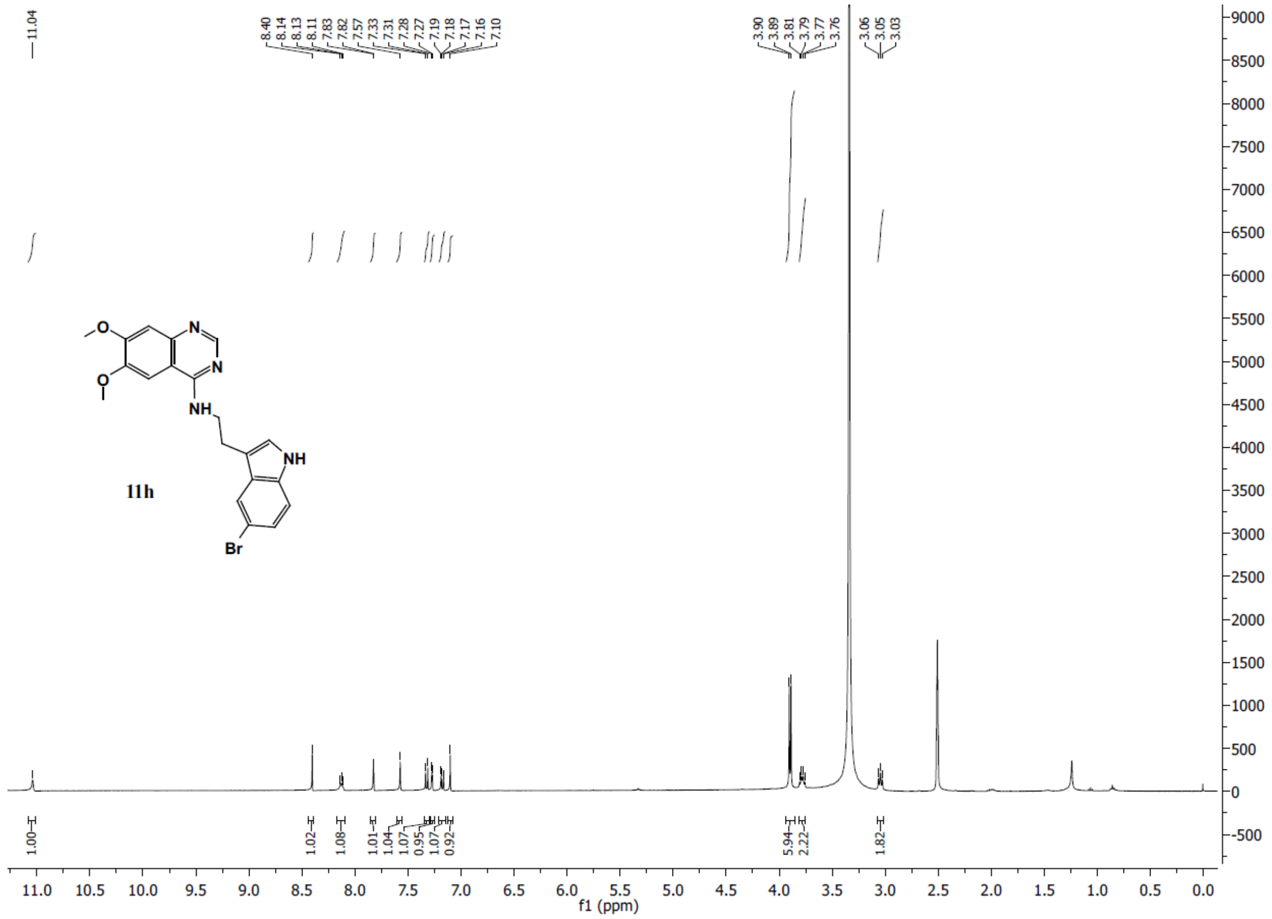
11.04
8.40 8.14 8.13 8.11 7.83 7.82 7.57 7.33 7.31 7.28 7.27 7.19 7.18 7.17 7.16 7.10
3.90 3.89 3.81 3.79 3.77 3.76
3.06 3.05 3.03
1.00 1.02 1.08 1.01 1.04 1.07 0.95 1.07 0.92 5.94 2.22 1.82
11h
NH
Br
f1 (ppm)

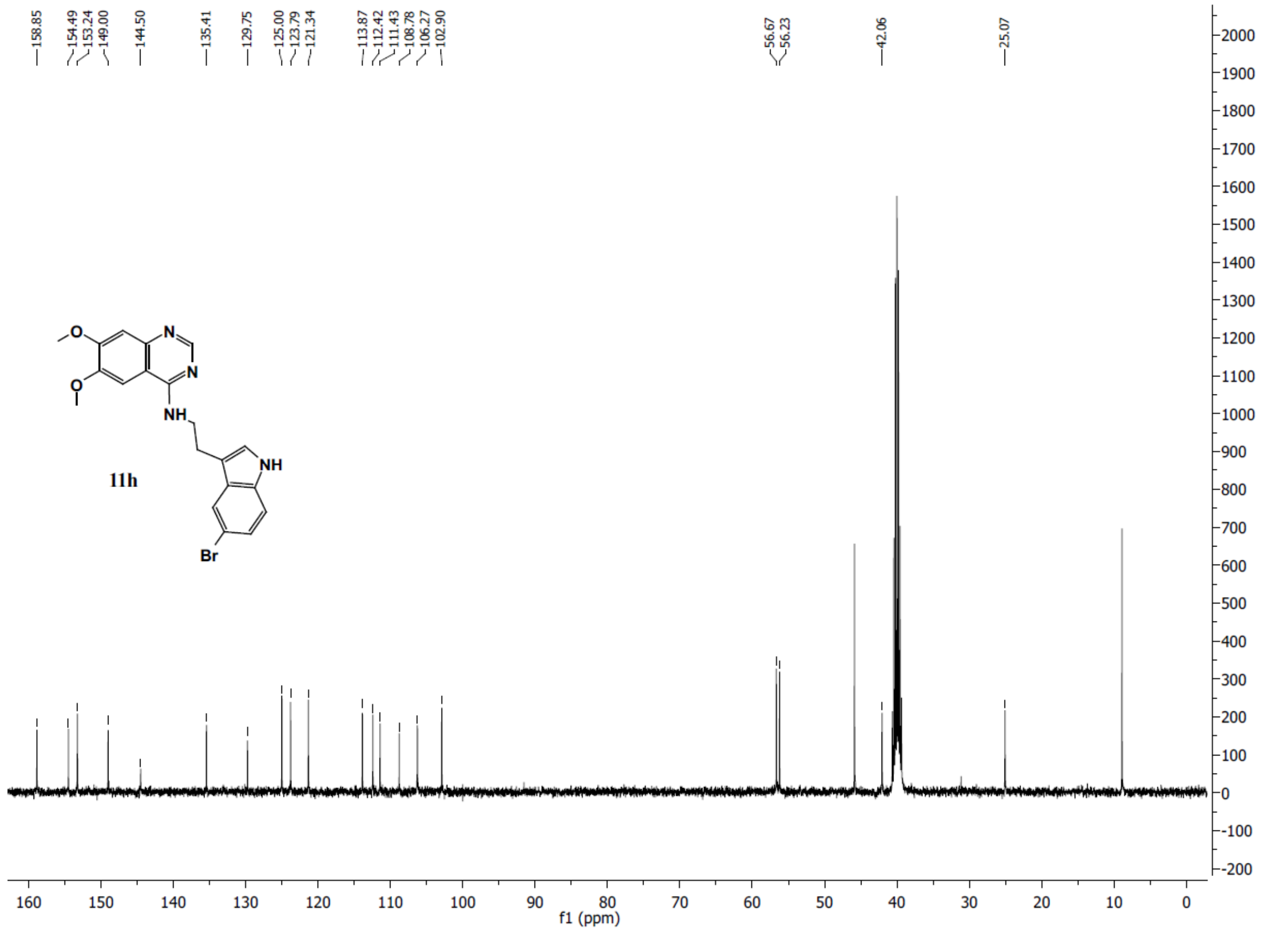
158.85 154.49 153.24 149.00 144.50 135.41 129.75 125.00 123.79 121.34
113.87 112.42 111.43 108.78 106.27 102.90
56.67 56.23
42.06
25.07
11h
NH
Br
f1 (ppm)

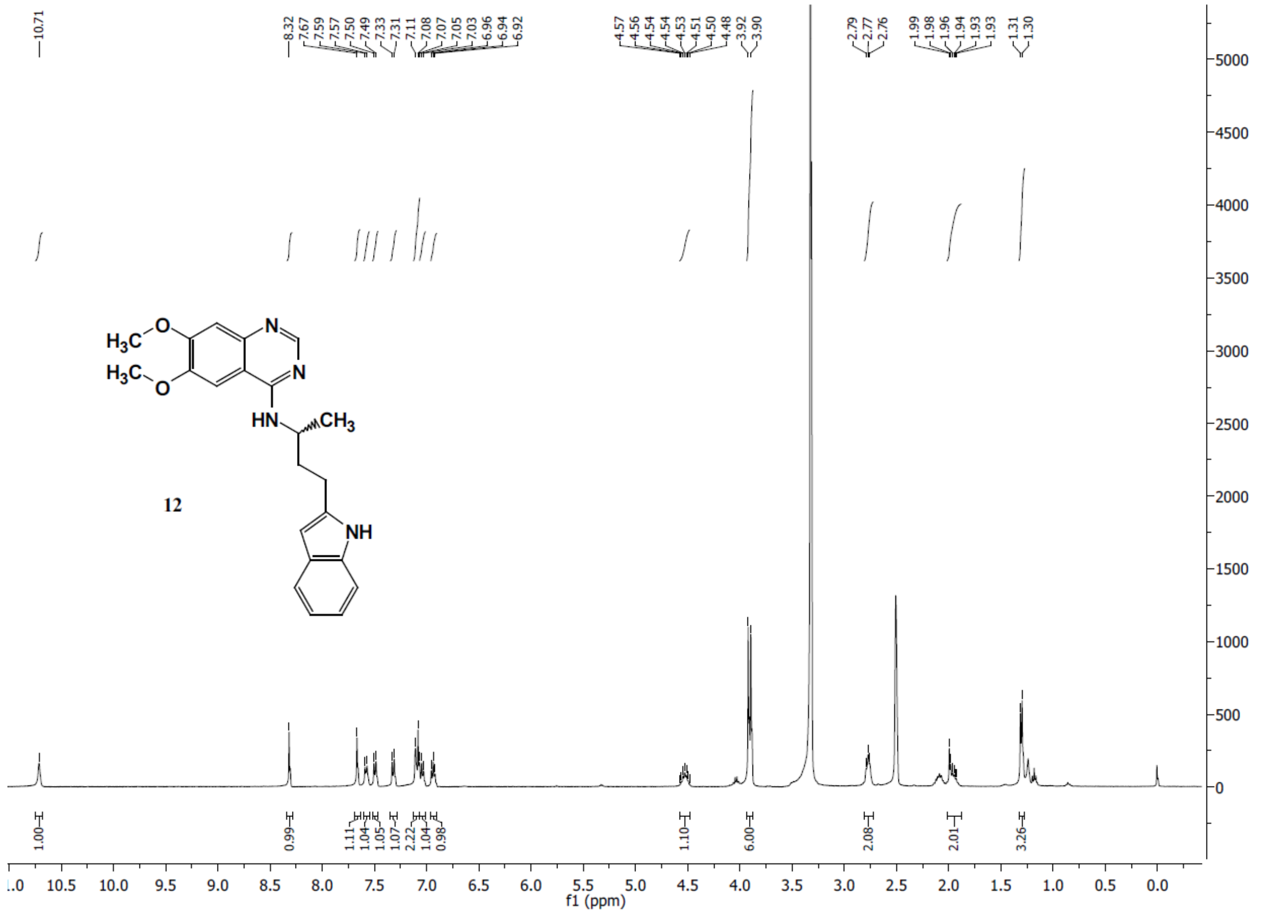

12

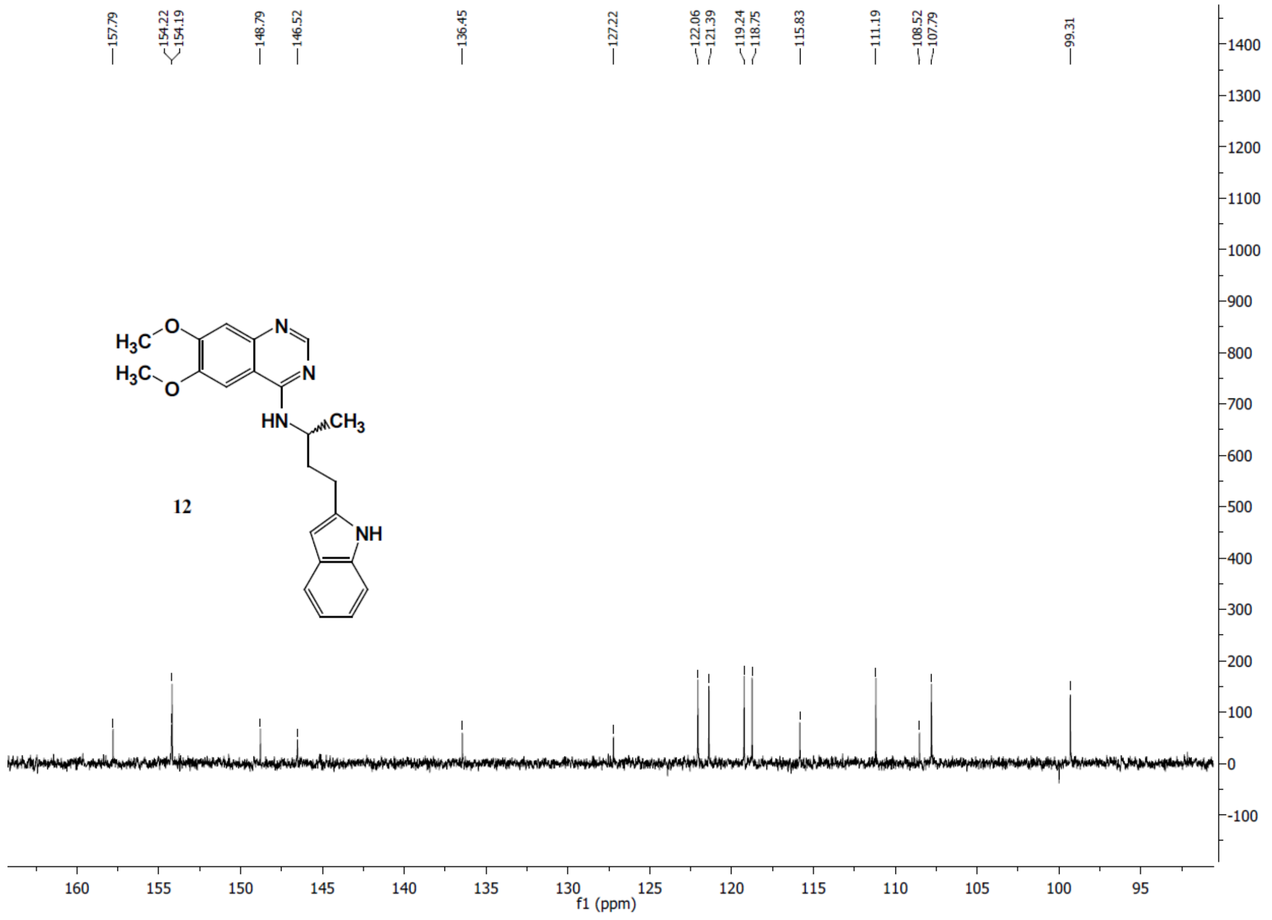

12

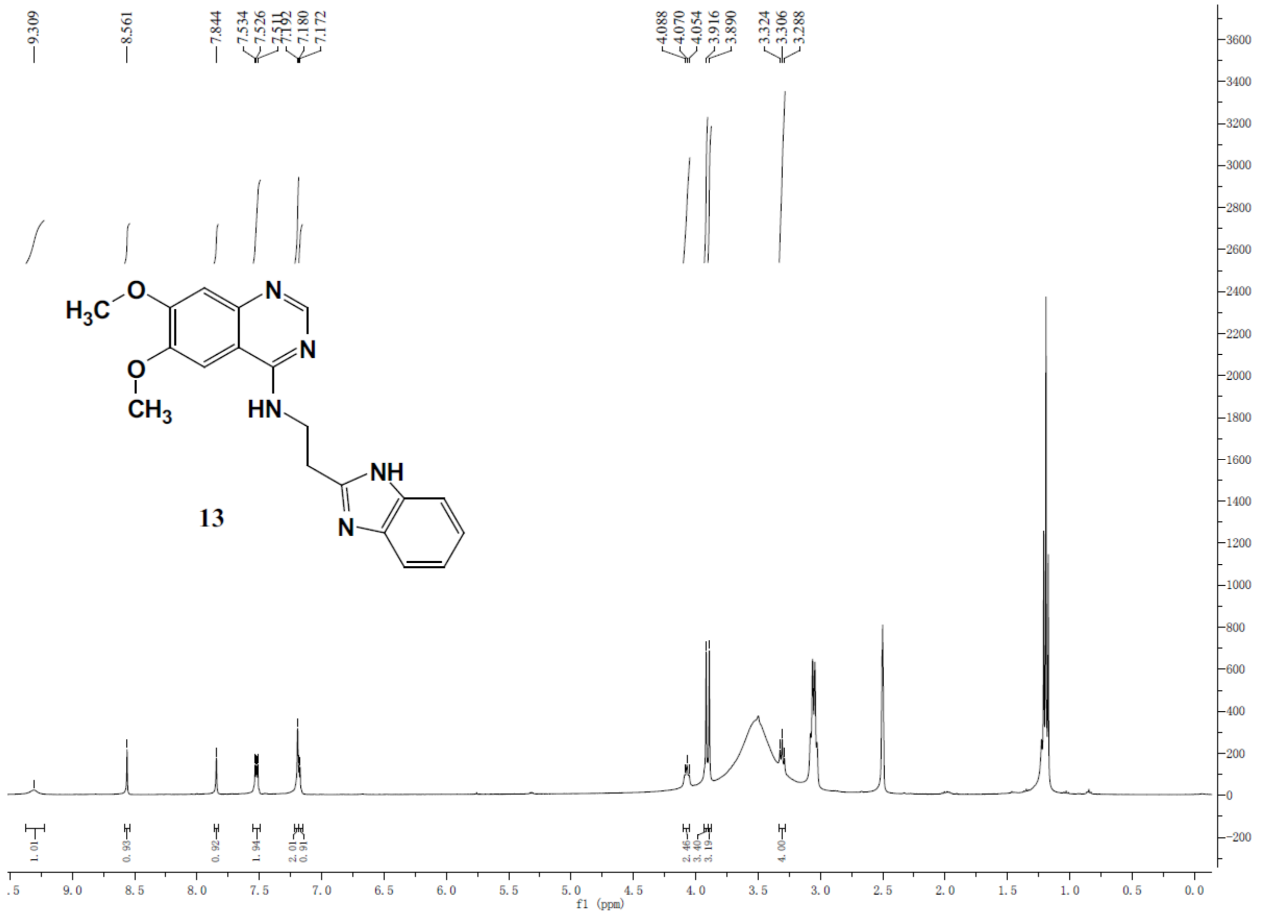
9.309
8.561
7.844
7.534
7.526
7.511
7.192
7.180
7.172
4.088
4.070
4.054
3.916
3.890
3.324
3.306
3.288
13
1.01
0.93
0.92
1.94
2.01
0.91
2.46
3.40
3.19
4.00
f1 (ppm)

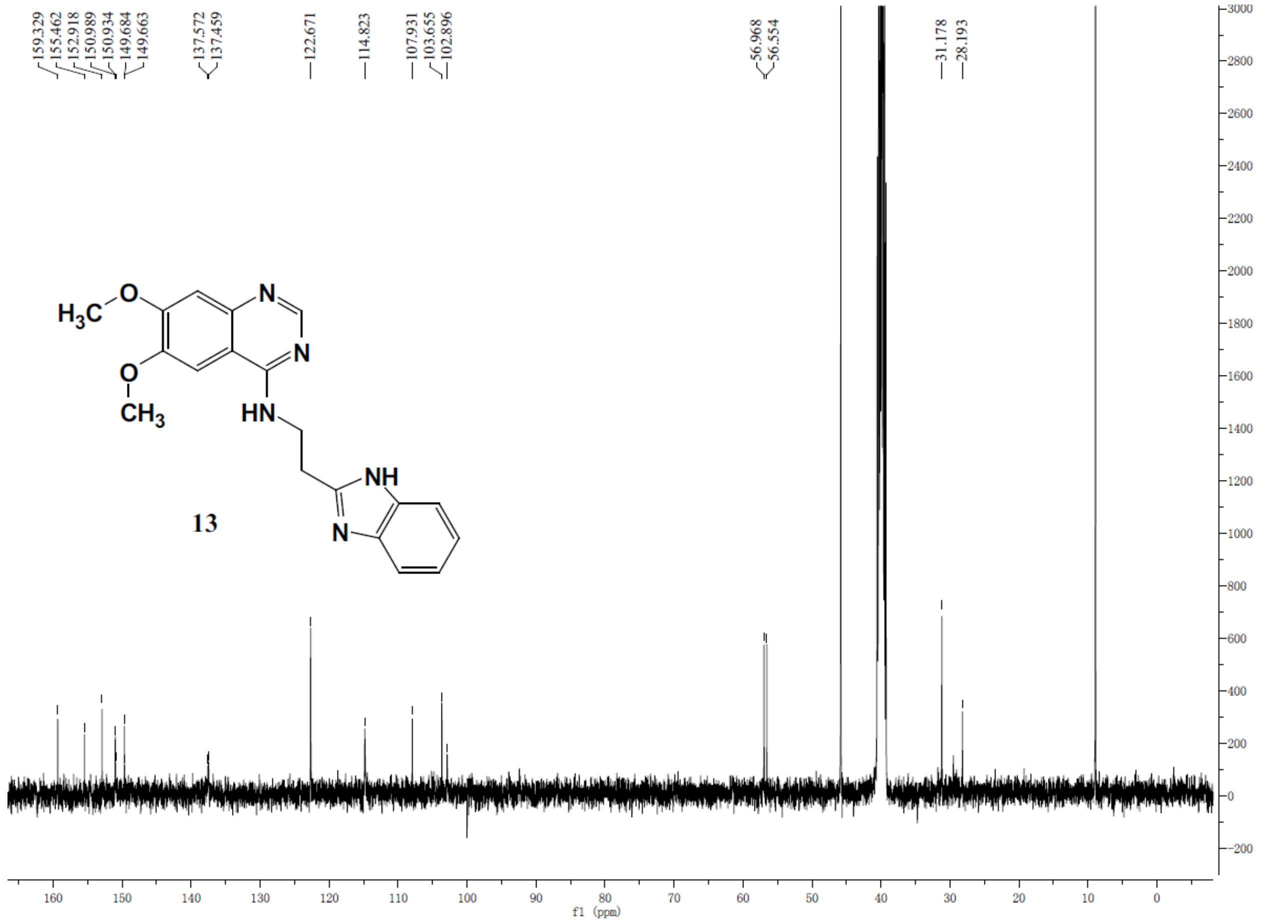
159.329
155.462
152.918
150.989
150.934
149.684
149.663
137.572
137.459
122.671
114.823
107.931
103.655
102.896
56.968
56.554
31.178
28.193
13
f1 (ppm)
